# DisP-seq reveals the genome-wide functional organization of DNA-associated disordered proteins

**DOI:** 10.1038/s41587-023-01737-4

**Published:** 2023-04-10

**Authors:** Yu-Hang Xing, Rui Dong, Lukuo Lee, Shruthi Rengarajan, Nicolò Riggi, Gaylor Boulay, Miguel N. Rivera

**Affiliations:** 1 Department of Pathology, Massachusetts General Hospital and Harvard Medical School, Charlestown, MA USA; 2 Broad Institute of MIT and Harvard, Cambridge, MA USA; 3 Center for Cancer Research, Massachusetts General Hospital and Harvard Medical School, Charlestown, MA USA; 4 Institute of Pathology, Centre Hospitalier Universitaire Vaudois, Faculty of Biology and Medicine, University of Lausanne, Lausanne, Switzerland; 5 Swiss Cancer Center Leman (SCCL), Centre Hospitalier Universitaire Vaudois, Faculty of Biology and Medicine, University of Lausanne, Lausanne, Switzerland

**Keywords:** Chromatin, Epigenomics, Cancer epigenetics, Transcriptional regulatory elements

## Abstract

Intrinsically disordered regions (IDRs) in DNA-associated proteins are known to influence gene regulation, but their distribution and cooperative functions in genome-wide regulatory programs remain poorly understood. Here we describe DisP-seq (disordered protein precipitation followed by DNA sequencing), an antibody-independent chemical precipitation assay that can simultaneously map endogenous DNA-associated disordered proteins genome-wide through a combination of biotinylated isoxazole precipitation and next-generation sequencing. DisP-seq profiles are composed of thousands of peaks that are associated with diverse chromatin states, are enriched for disordered transcription factors (TFs) and are often arranged in large lineage-specific clusters with high local concentrations of disordered proteins and different combinations of histone modifications linked to regulatory potential. We use DisP-seq to analyze cancer cells and reveal how disordered protein-associated islands enable IDR-dependent mechanisms that control the binding and function of disordered TFs, including oncogene-dependent sequestration of TFs through long-range interactions and the reactivation of differentiation pathways upon loss of oncogenic stimuli in Ewing sarcoma.

## Abstract (web summary)

Mapping DNA-associated disordered proteins across the genome uncovers gene regulatory patterns.

## Main

Intrinsically disordered regions (IDRs) in proteins lack a fixed tertiary structure under physiological conditions and have become increasingly recognized as having important functions^1^. IDRs can establish connections with complex protein–protein interaction networks^2^ and have been shown to contribute to essential cellular processes including signaling, transcription, RNA processing and cell cycle control^3–6^. IDRs can also promote cellular compartmentalization through the formation of biomolecular condensates^7,8^ and have been implicated in human disease, including neurodegeneration^3,9^ and cancer^10–12^.

**Extended Data Fig. 1:**
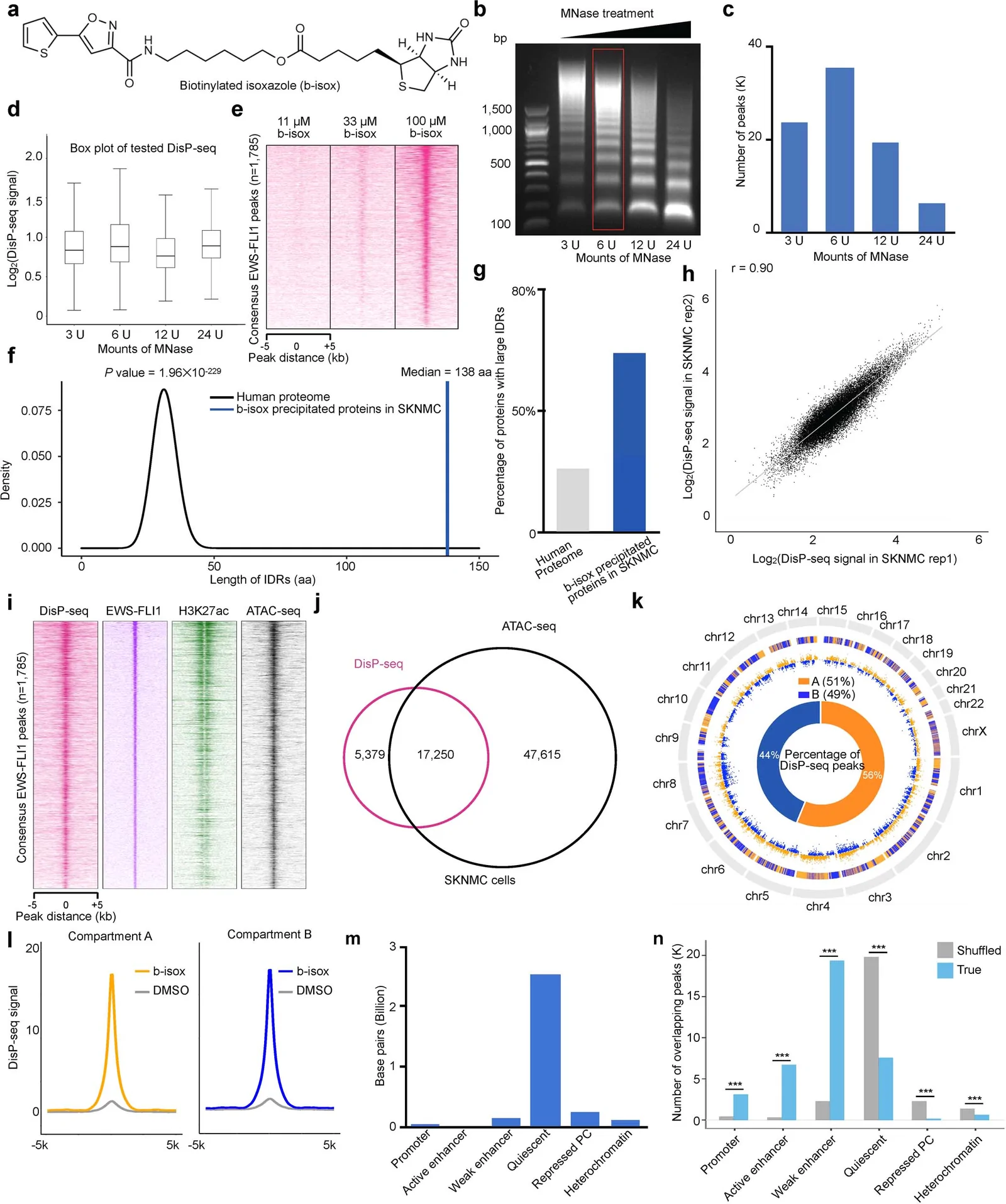
MNase titration and genome-wide distribution of DisP-seq in SKNMC cells. **a**, Chemical structure of b-isox. **b**, Electrophoresis of digested DNA from MNase titration experiments. The same amounts of DNA were loaded into a 1% agarose gel after MNase digestion. **c,d**, Total numbers **(c)** and signal intensities **(d)** of DisP-seq peaks observed with digestion at different MNase concentrations (n = 1 biologically independent experiment). Median value is shown as a line within the boxplot, which spans from the 25th to 75th percentiles. Whiskers indicate a 1.5× inter-quartile range. **e**, Heatmaps showing DisP-seq signal densities at EWS-FLI1 binding sites with different concentrations of b-isox in SKNMC cells. **f**, Graph showing the median length of IDRs found in b-isox precipitated nuclear proteins detected by mass spectrometry in SKNMC cells and permutations of equal numbers of proteins selected from the human proteome. The *P*-value was calculated by permutation test. **g**, Bar plot depicting the percentage of proteins with large IDRs in the human proteome and b-isox precipitated nuclear proteins detected by mass spectrometry in SKNMC cells. Permutation testing was used for *P*-value calculation. **h**, Scatter plot showing the Pearson correlation coefficient for peaks in two biological replicates of DisP-seq in SKNMC cells. **i**, Heatmaps showing DisP-seq, EWS-FLI1, H3K27ac ChIP-seq and ATAC-seq signal densities at EWS-FLI1 binding sites in SKNMC cells. **j**, Venn diagram depicting the overlap between DisP-seq and ATAC-seq peaks in SKNMC cells. **k**, Circos plots showing the distribution of DisP-seq signals in genomic compartments A (12,652 sites) and B (9,861 sites) in SKNMC cells. DisP-seq peaks are plotted as dots arranged on a y-axis based on intensity. **l**, Composite plots showing DisP-seq signal intensities in compartments A and B. **m**, Total numbers of base pairs assigned to different chromatin states by ChromHMM in SKNMC cells. **n**, Assessment of the significance of overlaps between DisP-seq peaks and different chromatin states defined by ChromHMM by OLOGRAM. The number of intersections is shown in blue and intersections calculated for shuffled regions are shown in gray. Statistical significance was calculated using a negative binomial model. ***p < 0.001. p_promoter_ < 1e-^320^, p_active enhancer_ < 1×10^-320^, p_weak enhancer_ < 1×10^-320^, p_quiescent_ < 1×10^-320^, p_repressed PC_ < 1×10^-320^, p_heterochromatin_ = 2.3×10^-285^.

Recent studies have linked IDR-containing proteins, including some proteins with low complexity domains (LCDs) composed of a limited subset of amino acids, to different types of gene regulatory functions. For example, some transcription factors (TFs) can mediate condensate formation to recruit transcriptional machinery to enhancers and promoters^5,6,10,13–15^. Components of repressive complexes can also drive phase separation to achieve chromatin compaction^16^ and linker histones can participate in phase separation and chromatin organization^17^. However, less is known about the coordinated role of these proteins in orchestrating genome-wide gene regulation programs. This is in large part due to the lack of methods to profile DNA-associated disordered proteins simultaneously across the genome. Current epigenomic methodologies designed to map DNA-associated proteins genome-wide, such as ChIP–seq, ORGANIC and CUT&RUN, require the use of antibodies and thus are limited to profiling previously defined proteins individually^18,19^. We sought to overcome this limitation by taking advantage of the physical and chemical properties of DNA-associated disordered proteins to produce genome-wide profiles in an antibody-independent manner. In particular, starting with pioneering studies by the McKnight laboratory, biotinylated isoxazole (b-isox; Extended Data Fig. 1a) has been shown to precipitate a large set of disordered proteins by forming microcrystals that nucleate the formation of β-strands in IDRs and LCDs^10,11,20–22^. These precipitates contain a complex mixture of disordered proteins and we reasoned that sequencing associated DNA fragments would produce genome-wide maps of the location of these proteins and enable us to study their involvement in gene regulation programs. Moreover, analysis of these profiles may also be used to identify disordered proteins linked to specific DNA-binding motifs, such as TFs with prominent IDRs, even when they are present in low amounts.

We now show that the combination of b-isox precipitation and next-generation sequencing (DisP-seq, disordered protein precipitation followed by DNA sequencing) can produce genome-wide profiles of endogenous DNA-associated disordered proteins simultaneously. Using this approach, we find that DNA-associated disordered proteins are distributed widely across the genome in complex and cell-type-specific patterns and can undergo large-scale reorganization with changes in cellular states. Moreover, prominent features in these landscapes, such as large clusters of DNA-associated disordered proteins (DisP islands, disordered protein-associated islands), are shaped by interactions between IDRs and have critical IDR-dependent regulatory functions in cellular differentiation and disease.

## Results

### DisP-seq signals are widely distributed in the genome

**Fig. 1:**
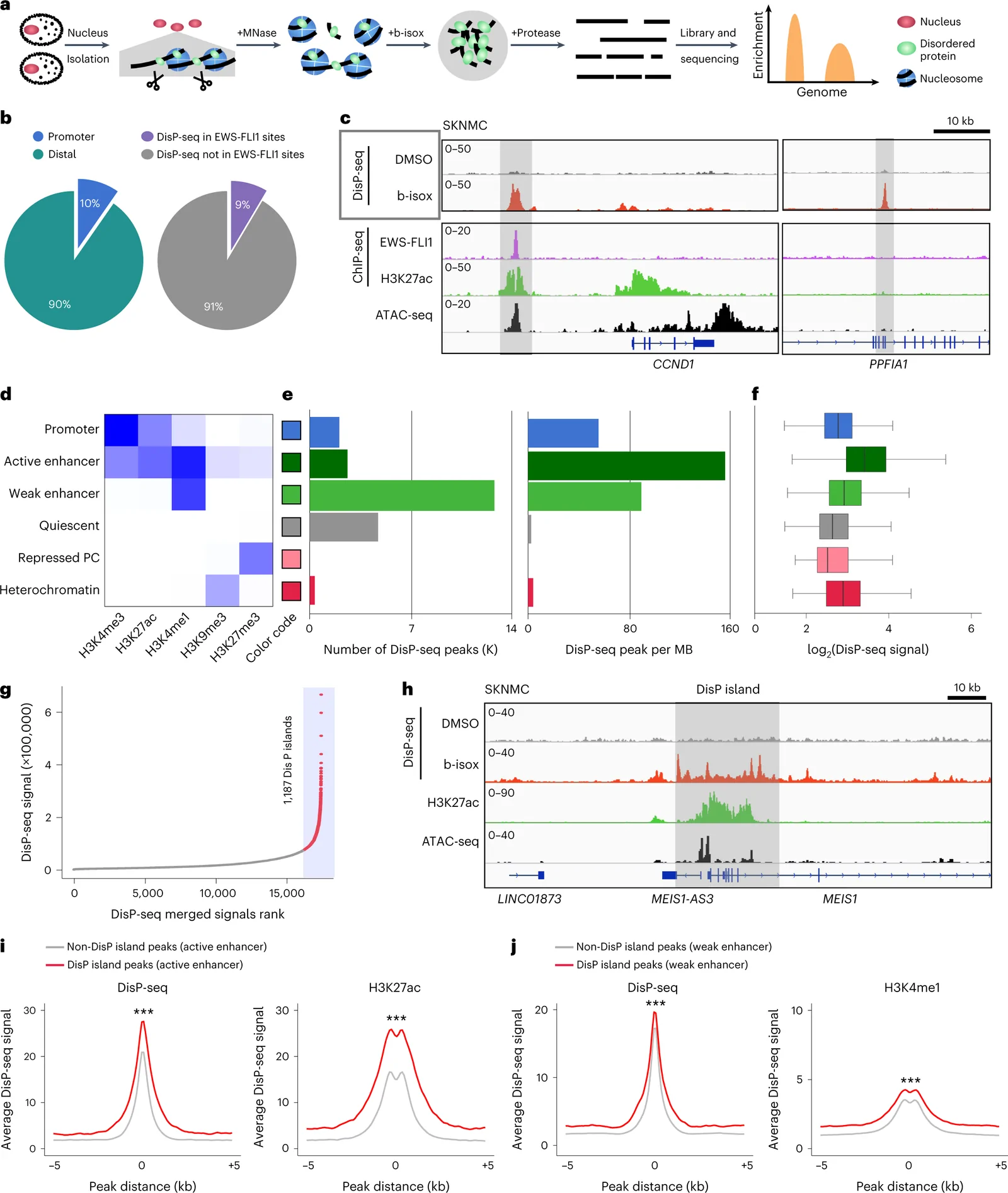
Genome-wide analysis of DisP-seq signals in Ewing sarcoma cells. **a**, Schematic of the DisP-seq assay. Nuclear extracts are treated by MNase digestion to release native endogenous proteins bound to DNA. Disordered proteins are precipitated by b-isox and the associated DNA is used to prepare libraries for sequencing. DMSO is used for controls. **b**, Pie chart depicting genome-wide distribution of all DisP-seq peaks (22,633, left) and the fraction of DisP-seq sites with EWS-FLI1 signals (right) in SKNMC cells. **c**, Representative examples of DisP-seq peaks and their chromatin context. Left, DisP-seq signals and DMSO controls at an EWS-FLI1 bound enhancer (highlighted in light gray) associated with *CCND1*. ChIP–seq signals for EWS-FLI1, H3K27ac and ATAC-seq signals are also shown. Right, DisP-seq signals and DMSO controls at a site without EWS-FLI1, H3K27ac ChIP–seq or ATAC-seq signals (*PPFIA1* locus, highlighted in light gray). **d**–**f**, DisP-seq peaks are classified into six categories by ChromHMM (**d**). The numbers of DisP-seq peaks and peaks per Mb in each category are shown in **e**. DisP-seq signal intensities for each category are shown in **f**, *n* = 2 biologically independent experiments. Median value is shown as a line within the boxplot, which spans from the 25th to 75th percentiles. Whiskers indicate a 1.5× interquartile range. **g**, Identification of DisP islands in SKNMC cells. DisP islands are defined as the population of merged signals above the inflection point of the curve (slope = 1). DisP-seq signals within 20 kb were merged for this analysis. **h**, Representative example of a DisP island with H3K27ac ChIP–seq and ATAC-seq signals (highlighted in light gray). **i**,**j**, Composite plots showing DisP-seq and H3K27ac or H3K4me1 signals for DisP peaks in active enhancers (**i**) and weak enhancers (**j**). DisP-seq peaks are divided into peaks inside or outside DisP islands. For each plot, ±5 kb regions centered on the DisP-seq peaks are shown (*x* axis). ***P < 0.001 (paired two-sided *t*-test). *P*_active enhancer DisP-seq_ = 1.01 × 10^−25^, *P*_active enhancer H3K27ac_ = 5.51 × 10^−34^, *P*_weak enhancer DisP-seq_ = 5.72 × 10^−39^, *P*_weak enhancer H3K4me1_ = 1.53 × 10^−53^.

We developed an assay capable of detecting endogenous DNA-associated disordered proteins through b-isox precipitation and DNA sequencing (DisP-seq; Fig. 1a). The assay involves (1) isolation of nuclei from unfixed cells, (2) digestion by micrococcal nuclease (MNase), (3) incubation with b-isox to precipitate disordered protein-DNA complexes and (4) DNA extraction and library preparation for next-generation sequencing. Our initial experiments were optimized by selecting the conditions that produced the strongest signal intensities and highest number of peaks in the Ewing sarcoma cell line SKNMC using different concentrations of MNase and b-isox (Extended Data Fig. 1b–e). We selected Ewing sarcoma, the second most common pediatric bone cancer, for these experiments because it is driven by the disordered oncogenic fusion protein EWS-FLI1 and serves as a paradigm for the role of IDRs in gene regulation^23,24^. In particular, the addition of the EWSR1 IDR to the ETS TF FLI1 allows the fusion protein to operate as a pioneer factor and induce active enhancers at GGAA microsatellite repeats in addition to binding canonical nonrepeat GGAA ETS binding sites^10,25^.

We also verified that the conditions used in our assay lead to the precipitation of disordered proteins as previously described^10,11,26^. We used mass spectrometry to analyze our b-isox precipitates and found that the median IDR annotation length in the MobiDB IDR database^27^ is markedly longer for precipitated nuclear proteins compared to a random size-matched subsampling of the human proteome (138 amino acids versus 31 amino acids; Extended Data Fig. 1f; *P* *=* 1.96 × 10^−229^). We also observed that most of the precipitated nuclear proteins contain large IDRs (greater than 100 amino acids). In contrast, large IDRs are found in 20% of the human proteome (Extended Data Fig. 1g). We expect that a subset of these proteins as well as other IDR-containing proteins that may be below the threshold of detection by mass spectrometry will be associated with DNA and will be visible in DisP-seq profiles.

DisP-seq of SKNMC cells showed strong peaks at 22,633 sites that were highly concordant between experiments (Pearson correlation 0.9, *p*-value < 10^-5^; Extended Data Fig. 1h). Annotation of these sites showed that 90% of DisP-seq peaks were associated with distal regions and 10% with gene promoters (Fig. 1b). We next analyzed signals at known binding sites for endogenous EWS-FLI1 in SKNMC cells as a positive control. This showed strong DisP-seq signals centered on EWS-FLI1 binding sites genome-wide, together with corresponding H3K27ac and ATAC signals indicating active and open chromatin (Extended Data Fig. 1i). DisP-seq is thus capable of yielding robust and specific peaks for a well-known disordered TF through antibody-independent chemical precipitation. Notably, EWS-FLI1 binding sites accounted for only a minority of DisP-seq peaks (9%; Fig. 1b), pointing to the detection of many other DNA-associated disordered proteins by this assay. Figure 1c shows examples of DisP-seq peaks associated with EWS-FLI1 (active enhancer with H3K27ac and ATAC-seq signals in the vicinity of *CCND1*) or without the presence of the fusion protein (a location without EWS-FLI1, H3K27ac or ATAC-seq signals).

**Extended Data Fig. 2:**
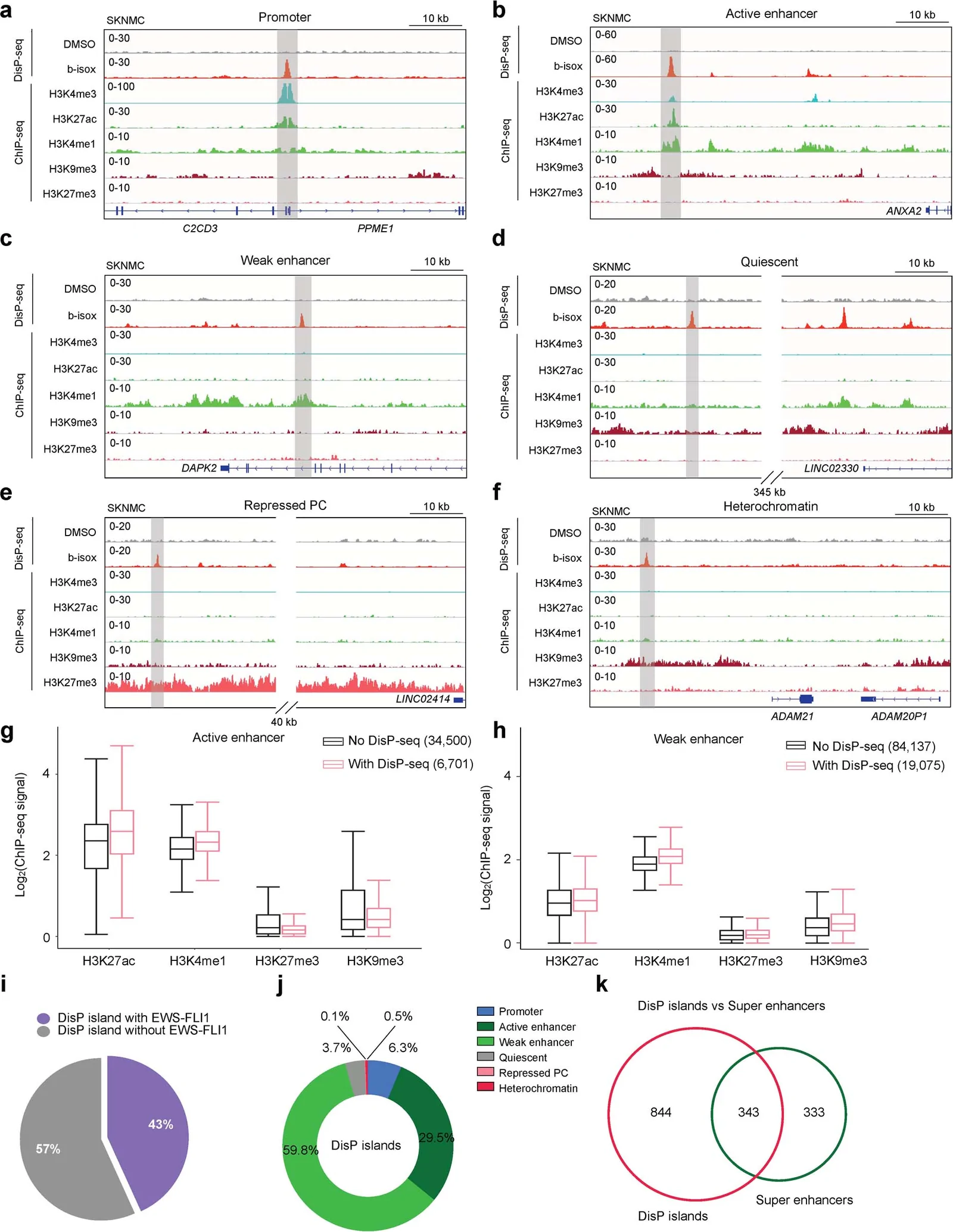
DisP-seq signals and DisP islands in different chromatin states defined by ChromHMM. **a-f**, Representative examples of DisP-seq peaks and associated chromatin marks in different chromatin states defined by ChromHMM: Promoter (**a**), Active enhancer (**b**), Weak enhancer (**c**), Quiescent (**d**), repressed polycomb (PC) (**e**) and heterochromatin (**f**). DisP-seq peaks are highlighted in light gray. **g,h**, Box plot showing levels of different histone modifications in active enhancers (**g**) and weak enhancers (**h**) with or without DisP-seq signals (n = 2 biologically independent experiments). Median value is shown as a line within the boxplot, which spans from the 25th to 75th percentiles. Whiskers indicate a 1.5× inter-quartile range. **i**, Pie chart depicting the fraction of DisP islands with EWS-FLI1 signals in SKNMC cells. **j**, Percentage of peaks of DisP islands in each ChromHMM category. **k**, Venn diagram depicting the overlap between DisP islands and Super enhancers in SKNMC cells.

A comparison with genome-wide chromatin accessibility ATAC-seq signals shows that DisP-seq peaks can occur at locations with either open (76%) or closed chromatin (24%). Moreover, DisP-seq peaks overlap only a minority of ATAC-seq peaks (Extended Data Fig. 1j), showing that DNA-associated disordered proteins are not evenly distributed in open chromatin. We also determined the distribution of DisP-seq signals in terms of large-scale 3D genomic compartments using ENCODE Hi-C maps for SKNMC cells. Fifty-six percent of DisP-seq peaks were present in the open A compartment and 44% in the closed B compartment, which is comparable to the ratio of compartment assignments in the genomic background of SKNMC cells (51% versus 49%; Extended Data Fig. 1k). We also observed the similar signal intensity levels in A and B compartments (Extended Data Fig. 1l). To more precisely determine which chromatin states were associated with DisP-seq signals, we assigned DisP-seq peaks to one of six states defined by ChromHMM using chromatin profiles for SKNMC cells^25,28^ (Fig. 1d and Extended Data Fig. 2a–f). In contrast to the genomic background (Extended Data Fig. 1m,n), DisP-seq peaks were most frequently found in weak enhancer regions, followed by active enhancer regions (Fig. 1e). The median levels of DisP-seq signals in different chromatin states were similar, with active enhancers having moderately higher levels overall (Fig. 1f). Because most DisP-seq peaks occur at enhancers, we also compared enhancers with or without DisP-seq peaks based on levels of different histone modifications. Active enhancers with DisP-seq peaks are associated with moderately higher H3K27ac and H3K4me1 signals (Extended Data Fig. 2g). Weak enhancers followed a similar pattern (Extended Data Fig. 2h). DisP-seq signals are thus associated with increased activation signals at enhancers, suggesting a potential relationship with increased regulatory activity. Taken together, our results show that DisP-seq peaks can be detected in the context of different types of genomic elements and are present in a subset of well-defined regulatory sites such as enhancers and promoters.

### DisP-seq reveals many large clusters of disordered proteins

Given that disordered proteins can engage in weak multivalent interactions through their IDRs^29^, we considered whether DisP-seq signals may occur as clusters in the genome. While the majority of DisP-seq signals occur as single peaks, we noted that certain locations contained clusters of DisP-seq signals spanning large genomic regions. To identify these regions, we grouped nearby DisP-seq peaks and ranked these groups by the total content of DisP-seq signals. This analysis showed a set of large DisP-seq clusters with the highest total DisP-seq signals, which we named DisP islands (Fig. 1g,h). DisP islands comprise 32.7% of the total DisP-seq signals in the genome and their median length is 21,975 bp compared to 981 bp for single DisP-seq peaks. Remarkably, almost half of DisP islands identified in SKNMC cells contain EWS-FLI1 peaks (Extended Data Fig. 2i), indicating that the presence of EWS-FLI1 is associated with neighboring signals for other disordered proteins.

To provide a detailed view of the chromatin landscape of DisP islands, we classified them according to our ChromHMM model for SKNMC cells. DisP islands are associated with various chromatin states and most are in enhancer regions (active enhancer and weak enhancer, Extended Data Fig. 2j). However, only a small subset of DisP islands overlapped superenhancers in SKNMC cells^30^ (Extended Data Fig. 2k). Because most DisP-seq peaks occur at enhancers, we next compared DisP-seq and histone mark signals at the enhancers associated DisP-seq peaks within or outside of DisP islands. At active enhancers, DisP island peaks are associated with higher DisP-seq signals and H3K27ac active enhancer marks compared to non-DisP island peaks (Fig. 1i). Similarly, we also observed higher DisP-seq signals and H3K4me1 basal enhancer marks in DisP island peaks of weak enhancers (Fig. 1j). These results suggest that DisP islands can provide an environment with high local concentrations of disordered proteins and increased chromatin marks linked to regulatory potential.

### DisP-seq peaks are enriched for specific TF motifs

**Fig. 2:**
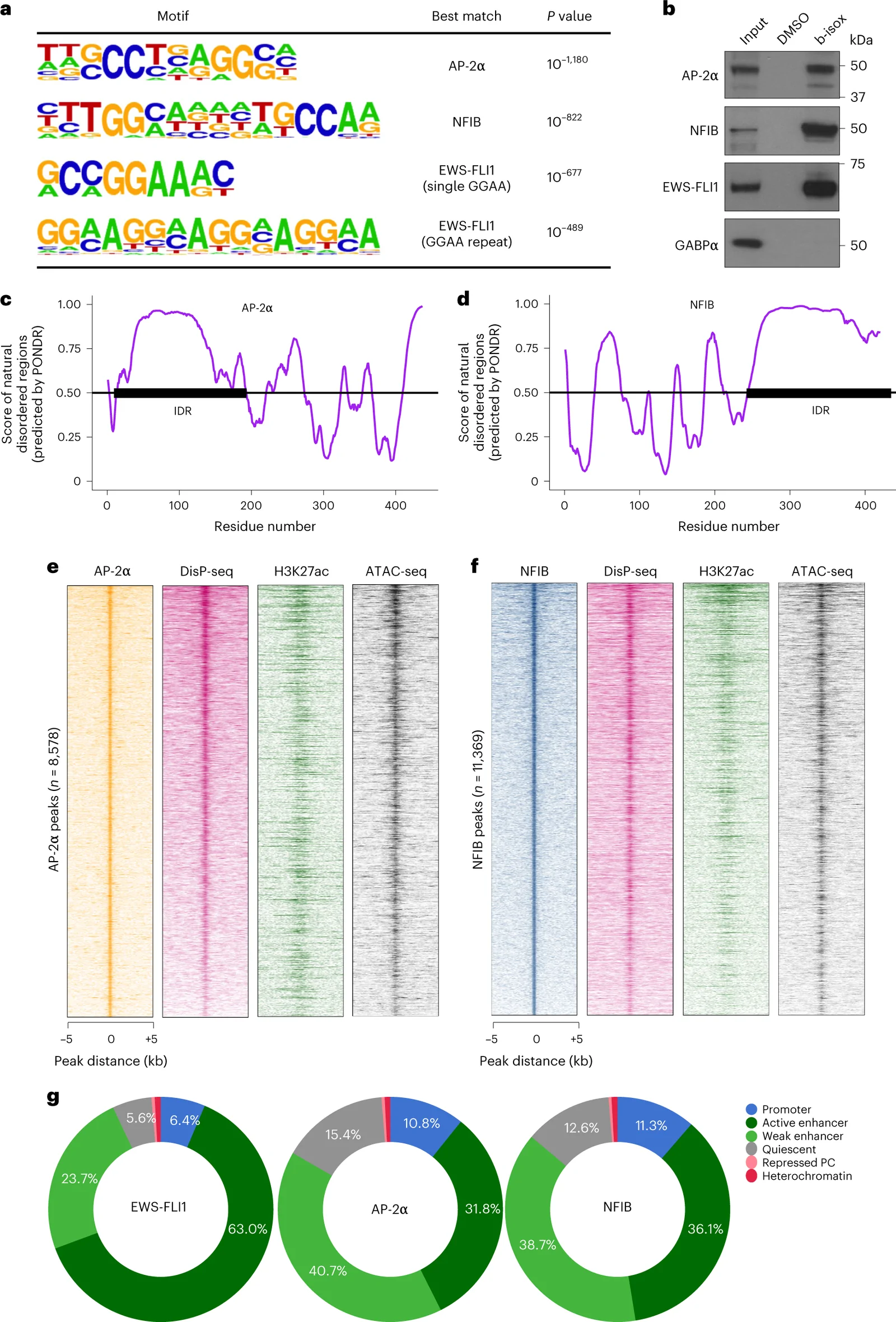
Identification of disordered TFs by DisP-seq in SKNMC cells. **a**, Motif enrichment analysis for DisP-seq signals in SKNMC cells. The top four motifs identified by HOMER are shown. **b**, Western blot after b-isox precipitation for the TFs identified in **a**. An ETS TF without a prominent disordered domain (GABPα) is shown as a control. **c**,**d**, Graphs showing intrinsic disorder for AP-2α (**c**) and NFIB (**d**). Intrinsic disorder scores were calculated by PONDR. IDRs were defined regions with PONDR scores equal to or higher than 0.5 for at least 50 amino acids. **e**,**f**, Heatmaps showing AP-2α ChIP–seq (**e**) and NFIB ChIP–seq (**f**) signals at DisP-seq sites in SKNMC cells (8,578 and 11,369 sites, respectively). For each heatmap, ±5 kb regions centered on the TF ChIP–seq peaks are shown. **g**, Percentage of peaks of different disordered TFs in each ChromHMM category. Source data

**Extended Data Fig. 3:**
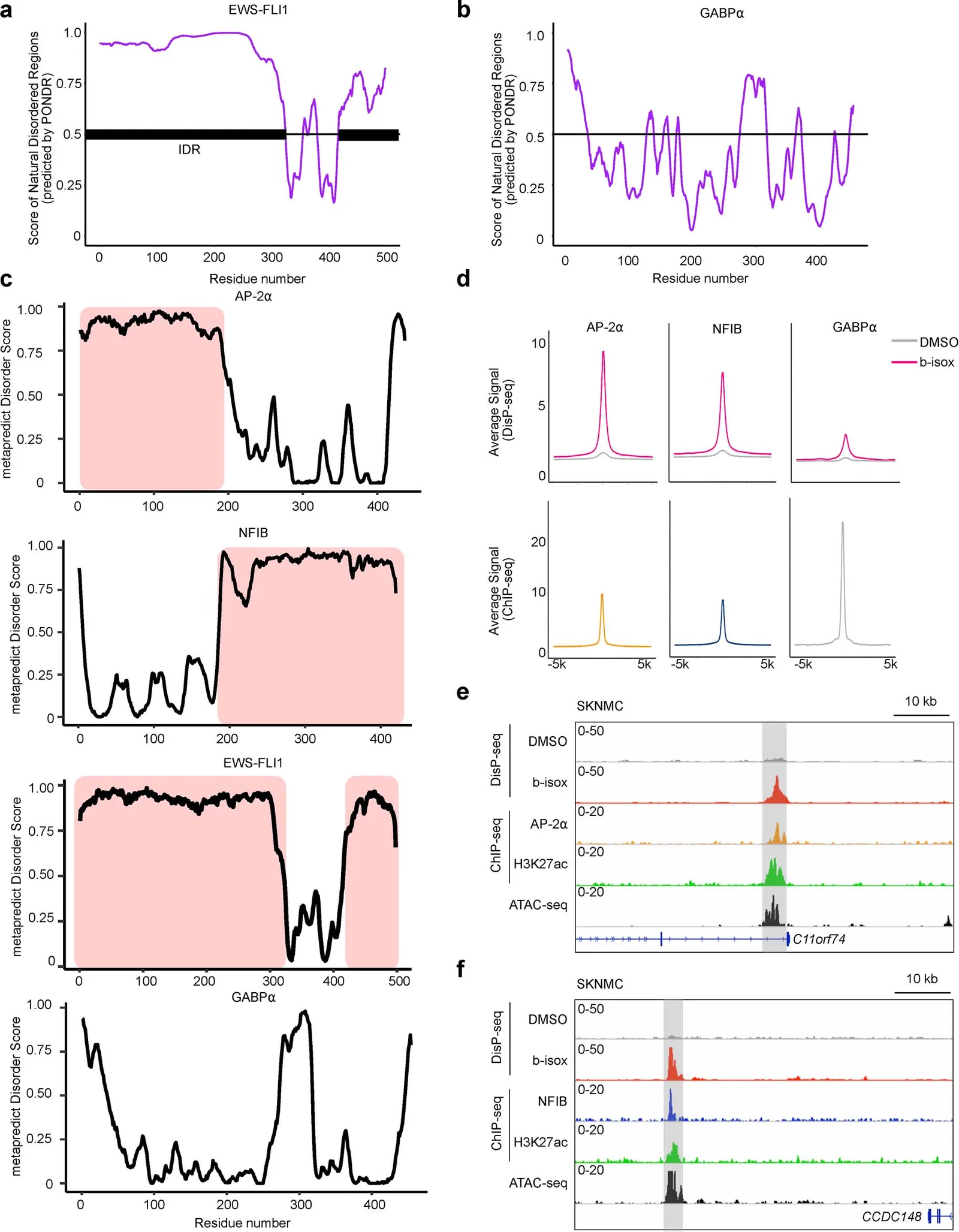
Prediction of IDRs in disordered transcription factors by PONDR and examples of DisP-seq signals at AP-2α and NFIB sites. **a,b**, Graphs showing intrinsic disorder for EWS-FLI1 (**a**) and GABPα (**b**). Intrinsic disorder scores were calculated by PONDR (VSL2 algorithm). IDRs were defined as regions with PONDR scores equal to or higher than 0.5 for at least 50 amino acids. **c**, Graphs showing intrinsic disorder for AP-2α, NFIB, EWS-FLI1 and GABPα. Intrinsic disorder scores were calculated by metapredict V2. IDRs were defined as regions with metapredict scores equal to or higher than 0.5 for at least 50 amino acids. **d**, Composite plots showing average levels of DisP-seq (top) and ChIP-seq signals (bottom) for the disordered transcription factors identified in Fig. 2a and for GABPα control sites in SKNMC cells. **e,f**, Representative examples of DisP-seq signals associated with AP-2α ChIP-seq peaks (*C11orf74* gene, **e**) and NFIB ChIP-seq peaks (*CCDC148* gene, **f**). DisP-seq peaks are highlighted in light gray.

We next performed an unbiased motif analysis for all DisP-seq peaks to identify enrichment for specific TFs at these sites in addition to EWS-FLI1. The top four motifs identified correspond to the TFs AP-2α, NFIB and EWS-FLI1 (single GGAA and GGAA repeat; Fig. 2a). To validate these results, we used western blotting to test precipitation of these TFs by b-isox. As expected, these experiments showed strong signals for AP-2α, NFIB and EWS-FLI1 (Fig. 2b). In contrast, signals were not observed for the wild-type endogenous ETS TF GABPα. These results match the prediction of IDRs by PONDR^31^. AP-2α, NFIB and EWS-FLI1 all have large IDRs compared to GABPα (Fig. 2c,d and Extended Data Fig. 3a,b). Given that several prediction methods for IDRs are available and may have different limitations^27,32–34^, we validated our results with a second approach (metapredict V2)^32^ and obtained similar results (Extended Data Fig. 3c).

To directly match DisP-seq signals with the TFs identified, we performed ChIP–seq for AP-2α and NFIB in SKNMC cells. Similar to our results for EWS-FLI1, AP-2α and NFIB sites were associated with strong DisP-seq signals centered on the corresponding TF-binding sites and with varying levels of H3K27ac and ATAC-seq (Fig. 2e,f). These signals were substantially higher than those at nonoverlapping GABPα sites despite strong GABPα ChIP–seq signals (Extended Data Fig. 3d). Examples of DisP-seq signals associated with AP-2α and NFIB peaks are shown in Extended Data Fig. 3e,f.

To explore the distribution of AP-2α and NFIB peaks identified by DisP-seq, we assigned these disordered TF peaks to six categories that we defined using ChromHMM (Fig. 2g). We found that most AP-2α and NFIB peaks are located at active and weak enhancers (73% and 75%, respectively). This is a similar distribution to EWS-FLI1 (87% at enhancers). In sum, DisP-seq signals are closely associated with TFs that contain prominent IDRs and can be used to identify these proteins in an unbiased antibody-independent manner.

### DisP signals are reorganized by changes in cellular states

We next sought to test whether changes in cellular states can lead to major differences in the distribution of DisP-seq signals. For this purpose, we measured DisP-seq signals in EWS-FLI1 depletion experiments, where the loss of the fusion protein is known to result in widespread changes in chromatin^25^. This process involves not only the loss of active signals at EWS-FLI1 binding sites but also the reactivation of normal mesenchymal differentiation programs that are typical of mesenchymal precursors, the putative cells of origin of Ewing sarcoma^10,25,35–37^. Mesenchymal differentiation in Ewing sarcoma cells with reduced levels of EWS-FLI1 has also been linked to increased migration, invasion and metastatic potential^23,24,38^.

**Fig. 3:**
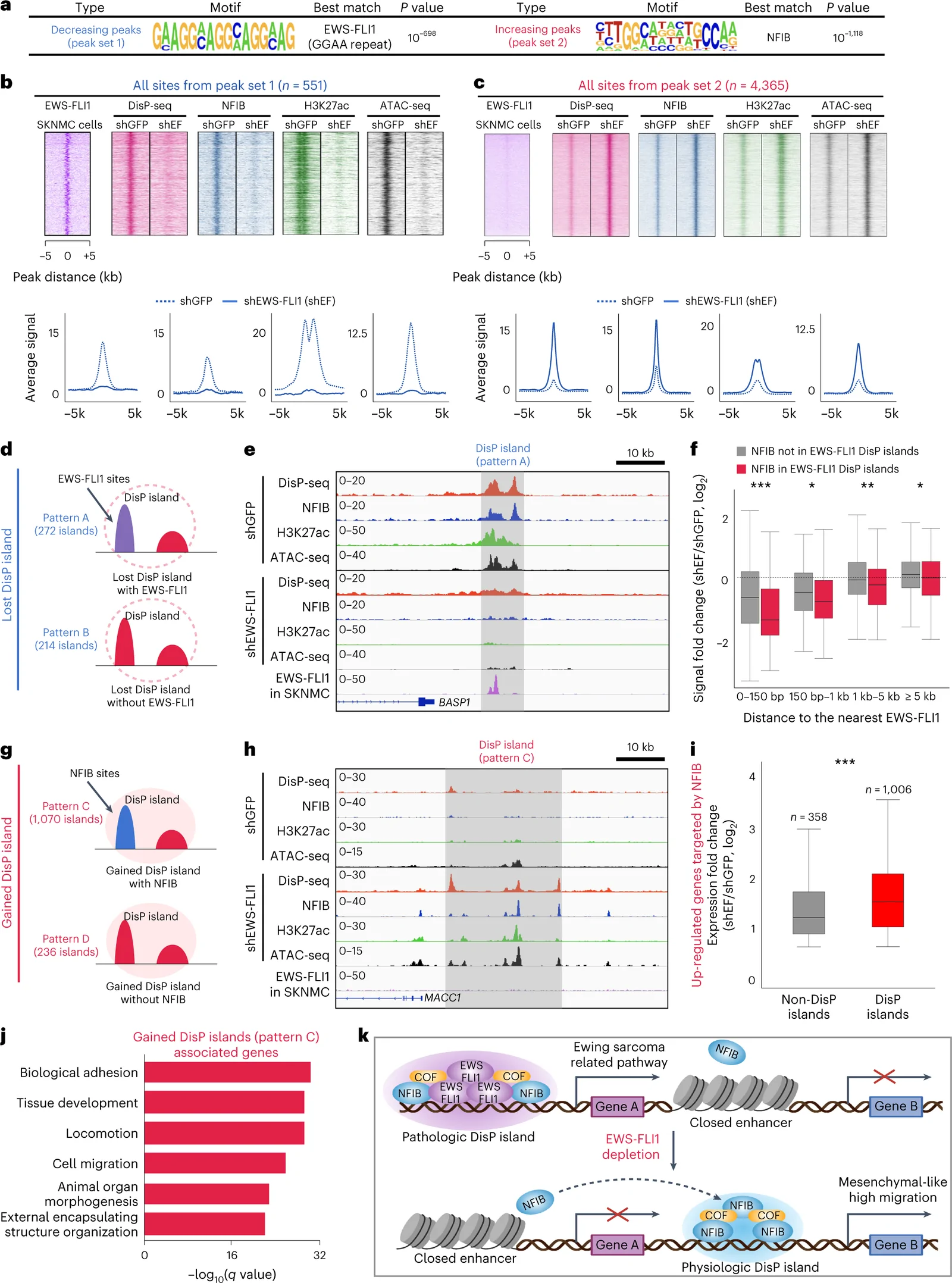
EWS-FLI1 depletion leads to reorganization of DisP islands in SKNMC cells. **a**, Top motifs for increasing and decreasing DisP-seq peaks (peak sets 1 and 2). **b**,**c**, Top—heatmaps depicting sites from peaks sets 1 (**b**) and 2 (**c**). DisP-seq, ChIP–seq and ATAC-seq signal intensities are shown. Bottom—composite plots for the same sites. **d**, Schematic of different patterns of DisP islands lost upon EWS-FLI1 depletion. DisP islands overlap with at least one (pattern A) or no EWS-FLI1 sites (pattern B). **e**, Representative example of DisP island classified as pattern A. **f**, Boxplot showing changes of NFIB signals after EWS-FLI1 depletion. Signals are grouped by their distance to EWS-FLI1 peaks and by whether they are in DisP islands associated with EWS-FLI1 (*n* = 2 biologically independent experiments). *P < 0.05, **P < 0.01 and ***P < 0.001 (two-sided *t*-test). Median value is shown as a line within the boxplot, which spans from the 25th to 75th percentiles. Whiskers indicate a 1.5× interquartile range. *P*_0–150 bp_ = 3.40 × 10^−18^, *P*_150–1 kb_ = 0.04, *P*_1k–5 kb_ = 0.004 and *P*_≥5 kb_ = 0.04. **g**, Schematic of different patterns of gained DisP islands upon EWS-FLI1 depletion. DisP islands that overlap with at least one (pattern C) or no increased NFIB peaks (pattern D). **h**, Representative example of DisP island classified as pattern C. **i**, Boxplot showing changes in expression for NFIB target genes after EWS-FLI1 depletion. Upregulated genes associated with NFIB sites are classified according to whether they are inside or outside of DisP islands (*n* = 2 biologically independent experiments). ***P < 0.001 (two-sided *t*-test). Median value is shown as a line within the boxplot, which spans from the 25th to 75th percentiles. Whiskers indicate a 1.5× interquartile range. *P* = 1.69 × 10^−07^ **j**, GO analysis of upregulated genes associated with gained DisP islands after EWS-FLI1 depletion. **k**, Schematic of DisP island reorganization after EWS-FLI1 depletion. In Ewing sarcoma cells, NFIB partially colocalizes with EWS-FLI1 in pathologic DisP islands. After EWS-FLI1 knockdown, NFIB signals are lost at GGAA repeats and gained at previously unoccupied NFIB sites to form physiologic DisP islands. The reorganization of DisP islands is associated with reactivation of normal mesenchymal differentiation programs in tumor cells. COF, cofactor.

**Extended Data Fig. 4:**
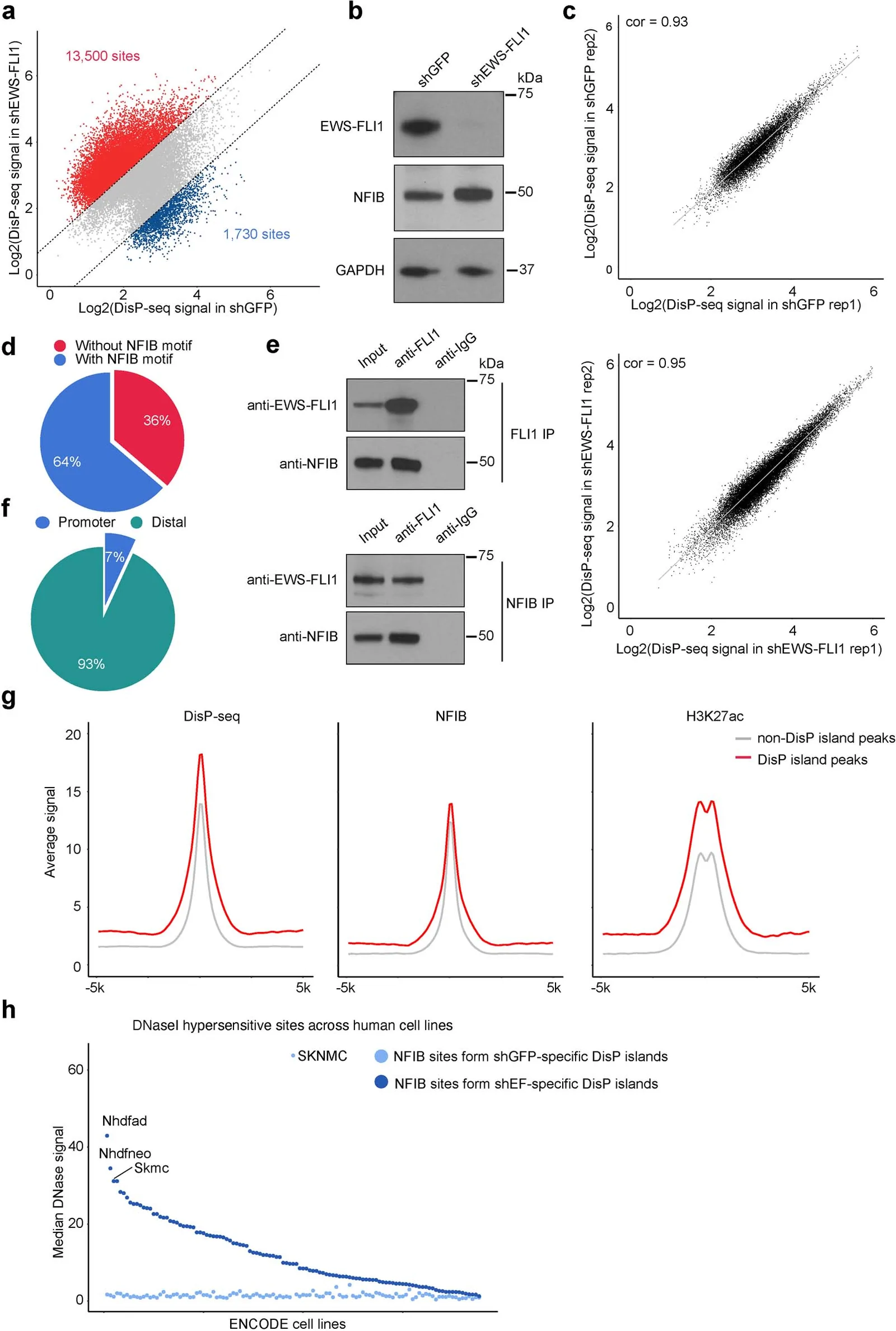
Reorganization of DisP-seq signals after EWS-FLI1 depletion. **a**, Scatter plot showing DisP-seq signals before and after depletion of EWS-FLI. Peaks with changes greater than two-fold are highlighted in red (increased) or blue (decreased). **b**, Western blot of EWS-FLI1, NFIB and GAPDH in EWS-FLI1 depleted cells and shGFP controls. GAPDH was used as a loading control. **c**, Scatter plot showing the correlation coefficient of two biological replicates of DisP-seq in shGFP (top) and shEWS-FLI1 (bottom) cells. **d**, Pie chart showing the proportion of GGAA microsatellites that contain the the half-site NFIB motif (TGGCA). **e**, Immunoprecipitation using an anti-FLI1 antibody (top) and anti-NFIB antibody (bottom) showing an interaction between EWS-FLI1 and NFIB in SKNMC cells. **f**, Pie chart showing the genome-wide distribution of increasing DisP-seq peaks after EWS-FLI1 knockdown. **g**, Composite plots showing intensities of DisP-seq, NFIB ChIP-seq and H3K27ac ChIP-seq signals from peak set 2 in DisP islands or outside of DisP islands. **h**, DNase I hypersensitivity profile comparison between NFIB sites that are specific to control SKNMC cells (shGFP) or EWS-FLI1 depleted cells (shEWS-FLI1) across 113 different cell types. Names are shown for selected cell types: Nhdfad (normal human dermal fibroblasts, adult), Nhdfneo (normal human dermal fibroblasts, neonatal), Skmc (human skeletal muscle cells) and SKNMC (Ewing sarcoma).

DisP-seq profiling showed 1,730 sites with decreased DisP-seq signals and 13,500 sites with increased signals after EWS-FLI1 depletion (Extended Data Fig. 4a–c). As expected, the top motif associated with decreased DisP-seq signals corresponds to EWS-FLI1 (Fig. 3a, GGAA repeats, peak set 1). Increasing peaks were strongly enriched for the disordered TF NFIB (*P* = 10^−1118^; Fig. 3a, peak set 2), which we initially identified in DisP-seq profiles of wild-type SKNMC cells and contains a large IDR (Fig. 2a,d). To verify the changes observed in DisP-seq peaks, we performed NFIB ChIP–seq in EWS-FLI1 depletion experiments and compared these results to DisP-seq signals, and ChIP–seq data for EWS-FLI1 and H3K27ac^25^. In agreement with our motif analysis, we found strong EWS-FLI1 ChIP–seq signals in peak set 1 and marked increases in NFIB ChIP–seq signals in peak set 2 (Fig. 3b,c). These peak sets were matched with corresponding decreases or increases in H3K27ac and ATAC-seq signals (Fig. 3b,c). Similar results were observed for DisP islands. A total of 486 DisP islands were lost and 1,306 DisP islands were gained upon EWS-FLI1 depletion. More than half of lost DisP islands were associated with EWS-FLI1 (Fig. 3d, pattern A), while 82% of gained DisP islands contain NFIB (Fig. 3g, pattern C). Our data thus show that DisP-seq signals can undergo substantial reorganization with changes in cellular states and that these profiles can be used to identify disordered TFs linked to these processes.

### DisP islands can enable sequestration of disordered TFs

In addition to these gained NFIB peaks, we also noted the presence of strong NFIB ChIP–seq signals near EWS-FLI1 sites before EWS-FLI1 depletion (Fig. 3b), including 270 of 272 EWS-FLI1 dependent DisP islands. This may be partly explained by the fact the half-site NFIB motif (TGGCA) is similar to GGAA and can be found within imperfect segments of GGAA microsatellites (64% of EWS-FLI1 bound GGAA repeat sites; Extended Data Fig. 4d) and by immunoprecipitation experiments that show an interaction between EWS-FLI1 and NFIB (Extended Data Fig. 4e). However, our data also shows that NFIB peaks within EWS-FLI1 DisP islands (pattern A) can be separated from the fusion protein by long distances (Fig. 3e) and prompted us to consider the relationship between EWS-FLI1 and nearby NFIB peaks in more detail. Remarkably, our data show that depletion of EWS-FLI1 strongly decreases NFIB binding (Fig. 3e,f) at both short and long distances between peaks (less than 150 bp and also between 150 bp and 1 kb). Moreover, NFIB peaks that are contained in EWS-FLI1-associated DisP islands are affected more strongly by the depletion of the fusion protein. This effect is less pronounced but still noticeable beyond 1 kb (Fig. 3f). Our data thus show effects of EWS-FLI1 on NFIB binding that are beyond the 50 bp range typically observed for cooperative binding^39^, suggesting that DisP islands can facilitate the coordinated binding of disordered TFs over large genomic regions.

Given the strong connection between NFIB and new DisP islands that appear after EWS-FLI1 depletion, we also characterized gene regulation programs associated with these events. NFIB is part of a family of TFs with roles in the development of various tissue types, including the differentiation of mesenchymal precursors^40^. Previous studies have also shown that NFIB can increase chromatin accessibility in small-cell lung cancer (SCLC) cells and stem cells^41,42^. Our results show that increases in NFIB binding mainly occur in distal regions (Extended Data Fig. 4f) and lead to the establishment of DisP islands (Fig. 3g,h). To explore the role of gained DisP islands in gene regulation, we examined upregulated genes near increasing NFIB signals after EWS-FLI1 KD. This analysis shows that increases in expression levels were more pronounced for genes associated with NFIB-containing DisP islands compared to NFIB peaks outside of DisP islands (Fig. 3i). Moreover, the signals of DisP-seq, NFIB and H3K27ac ChIP–seq are also higher in peaks located in gained DisP islands (Extended Data Fig. 4g). These results show that incorporation of NFIB into an environment with high local concentration of disordered proteins in DisP islands leads to enhanced effects on transcription programs.

Gene ontology (GO) analysis of potential target genes of these gained DisP islands shows a strong association with mesenchymal programs induced after EWS-FLI1 depletion (for example locomotion, adhesion and migration; Fig. 3j), pointing to a role for the reorganization of DisP islands in these processes. Moreover, sites with DisP-seq peaks in these DisP islands display high average DNase I signals in mesenchymal cell types profiled by ENCODE (113 human cell types, GSE29692), suggesting that they correspond to regulatory sites in this lineage^43^ (Extended Data Fig. 4h). In contrast, NFIB peak locations in DisP islands associated with EWS-FLI1 in SKNMC cells show low signals in all ENCODE cell types, suggesting that they are associated primarily with the Ewing sarcoma pathologic state (Extended Data Fig. 4h).

Taken together, our results show the reorganization of the DisP island landscape upon EWS-FLI1 depletion (Fig. 3k). In wild-type SKNMC cells, NFIB is partially sequestered by EWS-FLI1 in pathologic DisP islands, which are exclusively observed in the context of Ewing sarcoma. After EWS-FLI1 depletion, NFIB is released from these sites and relocates to NFIB binding sites linked to mesenchymal differentiation to establish physiologic DisP islands. In addition, the small increase in the level of NFIB protein after EWS-FLI1 KD may also contribute to the formation of these physiological DisP islands (Extended Data Fig. 4b). These changes are linked to the reactivation of latent mesenchymal differentiation programs in tumor cells through increased chromatin accessibility and enhancer activation.

### IDRs can mediate the incorporation of TFs into DisP islands

**Fig. 4:**
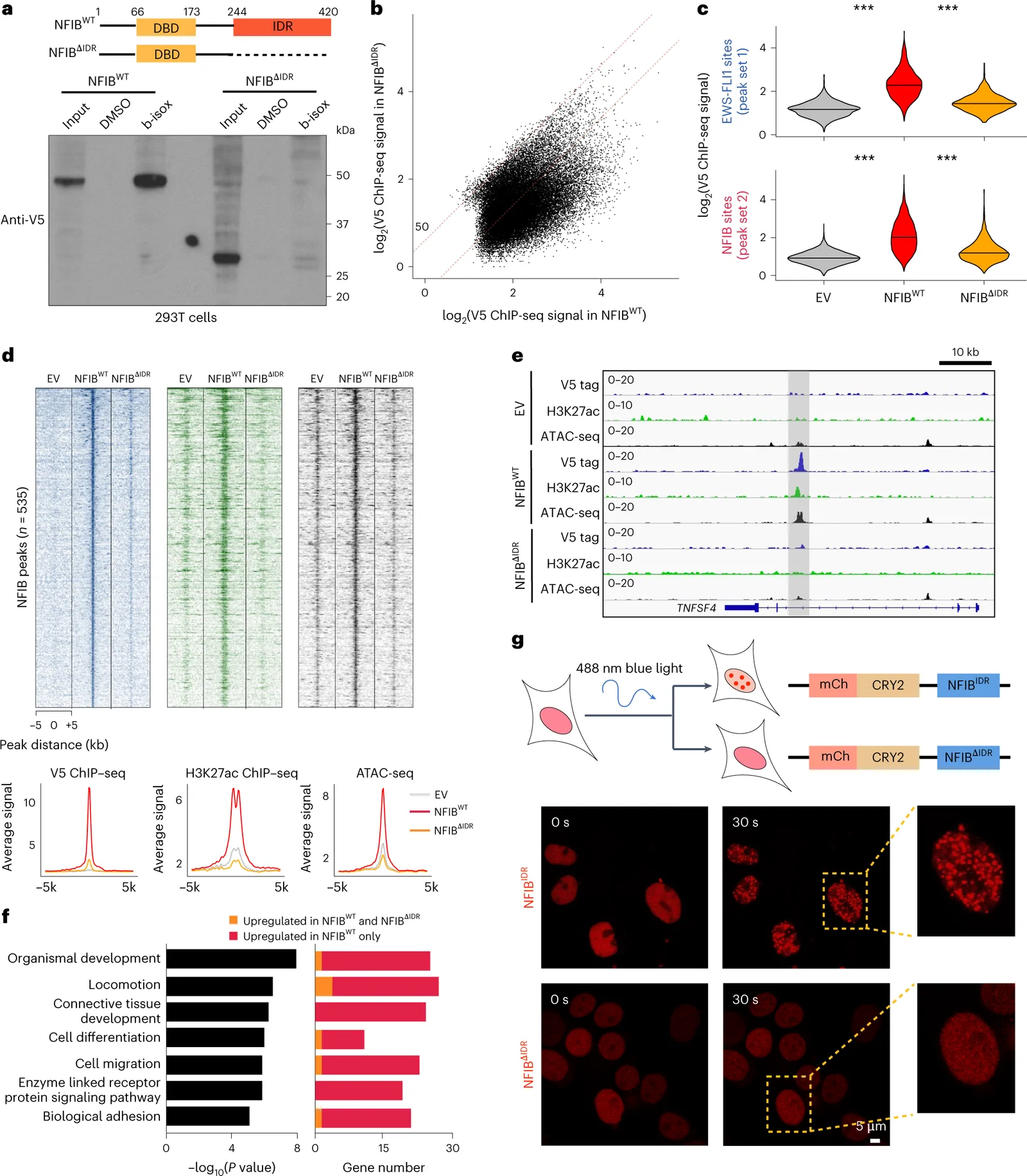
The NFIB IDR is critical for NFIB binding, function and formation of biomolecular condensates. **a**, Top—schematic comparing NFIB^WT^ and NFIB^ΔIDR^. Bottom—western blot after b-isox precipitation in 293 T cells transfected with NFIB^WT^ or NFIB^ΔIDR^. **b**, Scatter plot comparing V5 ChIP–seq signals for NFIB^WT^ and NFIB^ΔIDR^ cells. Red dashed lines indicate twofold differences. **c**, Violin plots showing V5 ChIP–seq signals in cells expressing exogenous NFIB at DisP-seq sites from peak set 1 (top) and peak set 2 (bottom). ***P < 0.001 (two-sided *t*-test). *P*_peak set 1(NFIB WT versus EV)_ = 5.34 × 10^−182^, *P*_peak set 1(NFIB WT versus NFIBΔIDR)_ = 5.52 × 10^−119^; *P*_peak set 2(NFIB WT versus EV)_ < 4.94 × 10^−324^, *P*_peak set 2(NFIB WT versus NFIBΔIDR)_ < 4.94 × 10^−324^. **d**, Heatmaps (top) and composite plots (bottom) depicting V5, H3K27ac ChIP–seq and ATAC-seq signal intensities in empty vector (EV), NFIB^WT^ and NFIB^ΔIDR^ cells. A total of 535 NFIB sites with increasing NFIB, H3K27ac and ATAC-seq signals in both EWS-FLI1 knockdown and NFIB overexpression experiments are shown. Each heatmap shows ±5 kb regions centered on NFIB peaks. **e**, Representative example of differences in signals between NFIB^WT^ and NFIB^ΔIDR^ at the *TNFSF4* locus. The NFIB^WT^ peak region is highlighted in light gray. **f**, Left—GO analysis of upregulated genes upon expression of NFIB. Right, number of genes in each GO category for NFIB^WT^ and NFIB^ΔIDR^. **g**, Top—schematic of the optoDroplet assay. mCherry-CRY2 was fused with the IDR of NFIB or with NFIB^ΔIDR^. Cells expressing these constructs were tested for droplet formation after exposure to 488 nm blue light. Bottom—representative images of NFIB^IDR^-mCherry-CRY2 (top) and NFIB^ΔIDR^ -mCherry-CRY2 (bottom) fusion proteins expressed in 293 T cells. Cells were stimulated with a 488 nm laser for 30 s before imaging.

Given the strong association between NFIB and changes in DisP-seq signals, we next considered whether the IDR of NFIB has a role in the binding and function of this TF. NFIB contains a large IDR in its C-terminal region (Fig. 2d) and we generated a mutant lacking this domain (NFIB^ΔIDR^; Fig. 4a). We first compared wild-type NFIB (NFIB^WT^) and NFIB^ΔIDR^ by b-isox precipitation and, as expected, only NFIB^WT^ signals were detected by Western blot in the precipitated fraction (Fig. 4a).

**Extended Data Fig. 5:**
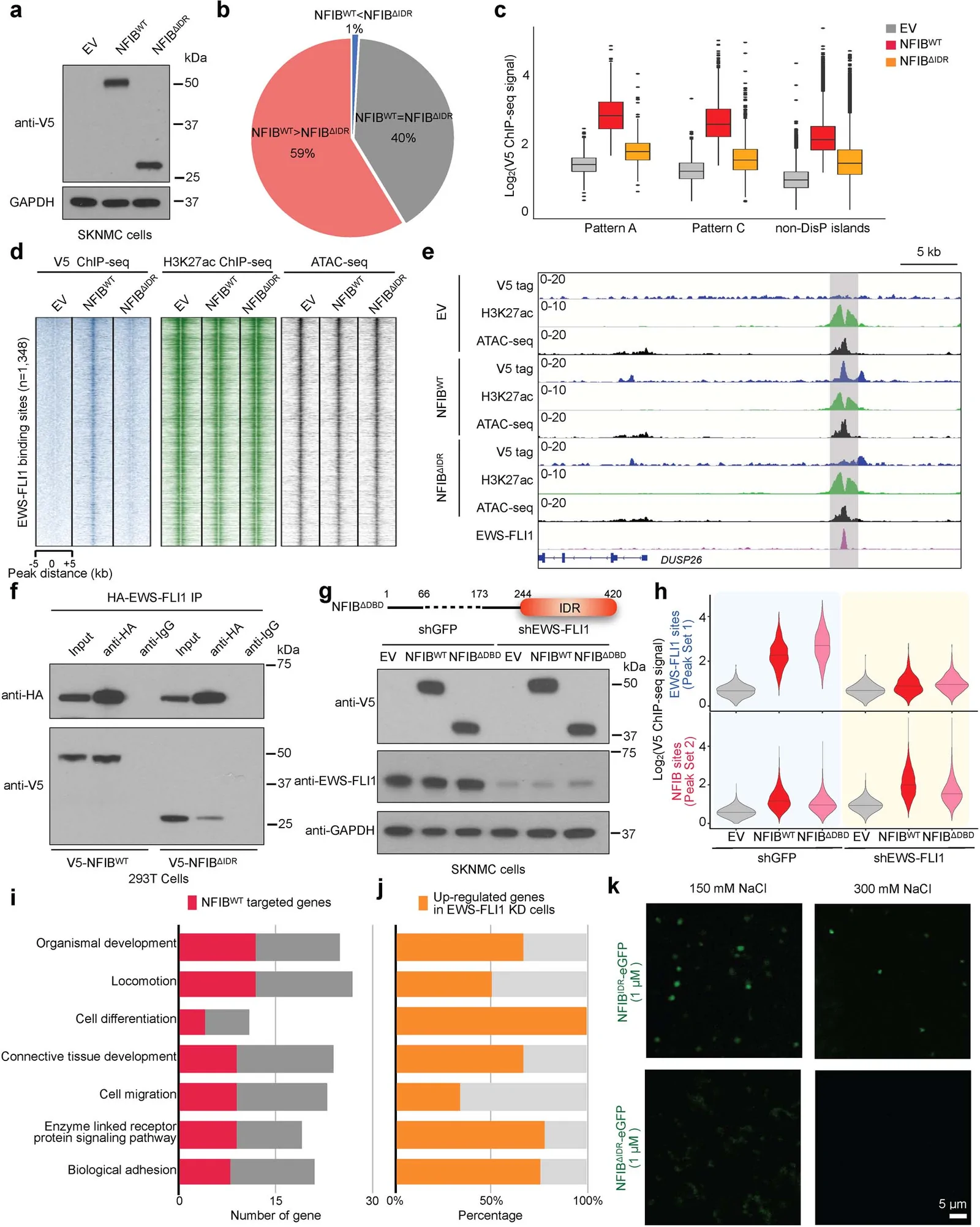
The NFIB IDR is required to activate mesenchymal differentiation genes in Ewing sarcoma cells. **a**, Western blot of V5 and GAPDH in SKNMC cells infected with empty vector (EV), NFIB^WT^ and NFIB^ΔIDR^. GAPDH was used as a loading control. **b**, Pie chart quantifying signals after introduction of NFIB^WT^ or NFIB^ΔIDR^ in SKNMC cells. Most peaks have stronger signals (at least 1.5-fold) in NFIB^WT^ compared to NFIB^ΔIDR^. **c**, Box plot showing changes of V5 signals inside or outside DisP islands (Patterns A and C) after overexpression of NFIB^WT^ and NFIB^ΔIDR^ (n = 2 biologically independent experiments). Median value is shown as a line within the boxplot, which spans from the 25th to 75th percentiles. Whiskers indicate a 1.5× inter-quartile range. **d**, Heatmaps depicting V5 tag, H3K27ac ChIP-seq and ATAC-seq signal intensities at EWS-FLI1 binding sites in EV, NFIB^WT^ and NFIB^ΔIDR^ cells. **e**, Representative example of NFIB binding at a GGAA microsatellite repeat enhancer associated with the *DUSP26* gene. The enhancer region is highlighted in light gray. **f**, Western blot of immunoprecipitation experiment using an anti-HA antibody in HA-EWS-FLI1 and V5- NFIB^WT^ or NFIB^ΔIDR^ double transfected 293 T cells. **g**, Top: Schematic comparing NFIB^WT^ and NFIB^ΔDBD^. Bottom: Western blot of V5, EWS-FLI1 and GAPDH in EWS-FLI1 KD cells infected with empty vector (EV), NFIB^WT^ or NFIB^ΔDBD^. GAPDH was used as a loading control. **h**, Violin plots showing V5 ChIP-seq signals in shGFP controls and EWS-FLI1 KD cells expressing exogenous NFIB^WT^ and NFIB^ΔDBD^. Signals were calculated at DisP-seq sites from peak set 1 (top) and peak set 2 (bottom). **i**, GO analysis of genes upregulated upon introduction of NFIB^WT^ (gray and red combined). The subset of genes that are NFIB^WT^ target genes is shown in red. Target genes were defined as the nearest genes to V5-NFIB^WT^ ChIP-seq peaks. **j**, Graph showing the percentage of genes in each GO category in (**g**) that are also increased following EWS-FLI1 knockdown (orange). **k**, *In vitro* droplet formation assay with recombinant fusion proteins. NFIB^IDR^-eGFP (IDR of NFIB fused to eGFP). NFIB^ΔIDR^-eGFP (NFIB lacking IDR fused to eGFP).

We next introduced V5-tagged NFIB^WT^ and NFIB^ΔIDR^ into SKNMC cells (Extended Data Fig. 5a) and performed V5 ChIP–seq to test the binding patterns of these proteins. A genome-wide comparison showed that most binding sites had signals that were either similar for both NFIB^WT^ and NFIB^ΔIDR^ or substantially higher for NFIB^WT^ (40% and 59%, respectively; Fig. 4b and Extended Data Fig. 5b). In particular, median NFIB^WT^ signals were at least twofold higher in peak set 1 and peak set 2 (Fig. 4c). Furthermore, exogenous wild-type NFIB is more effectively incorporated into pattern A and pattern C DisP islands compared to NFIB^ΔIDR^ while this difference is less apparent outside of DisP islands (Extended Data Fig. 5c). These results show that the IDR of NFIB is required for incorporation into DisP islands, pointing to a role of IDR mediated interactions in establishing coordinated TF-binding patterns.

The differences between NFIB^WT^ and NFIB^ΔIDR^ observed at EWS-FLI1 bound GGAA repeats in peak set 1 show that NFIB occupancy at these sites is highly dependent on its IDR. We did not observe changes in ATAC-seq or H3K27ac signals after NFIB^WT^ and NFIB^ΔIDR^ overexpression at GGAA repeats (Extended Data Fig. 5d,e), consistent with the notion that EWS-FLI1 is the primary activator of these elements. Given the IDR-dependent difference in NFIB recruitment, we considered whether the NFIB IDR may facilitate binding at GGAA repeats through interactions with EWS-FLI1. To test for this possibility, we performed co-immunoprecipitation after expressing tagged EWS-FLI1 (HA-EWS-FLI1) and V5-NFIB^WT^ or V5-NFIB^ΔIDR^ in 293 T cells. These experiments showed a stronger interaction for NFIB^WT^ compared to NFIB^ΔIDR^ (Extended Data Fig. 5f), suggesting that interactions mediated by the NFIB IDR may contribute to the localization of NFIB to pathologic DisP islands. To further test these contributions, we also compared V5-NFIB^WT^ to an NFIB mutant lacking the DNA-binding domain (V5-NFIB^ΔDBD^; Extended Data Fig. 5g) by ChIP–seq. Remarkably, both NFIB^WT^ and NFIB^ΔDBD^ produced strong signals in peak set 1 (Extended Data Fig. 5h), suggesting that the NFIB IDR can be sequestered at EWS-FLI1 GGAA repeat sites without a DNA-binding domain. This effect was dependent on EWS-FLI1 because it was not observed in cells with knockdown of the fusion protein. Peak set 2 sites also showed increased signals for NFIB^ΔDBD^ (Extended Data Fig. 5h). This effect was more noticeable upon EWS-FLI1 depletion when endogenous NFIB is relocated to these sites. Together, these data support the conclusion that the IDR of NFIB has an important role in sequestration by EWS-FLI1 and are in keeping with recent studies showing that IDRs can affect DNA-binding site selection by TFs^44,45^.

NFIB^WT^ signals were also markedly higher at NFIB sites associated with differentiation after EWS-FLI1 knockdown (Fig. 4d,e). In keeping with this finding, ATAC-seq and H3K27ac signals were increased in NFIB^WT^ cells at these sites while they remained unchanged in NFIB^ΔIDR^ cells (Fig. 4d,e). A comparison of RNA-seq profiles in NFIB^WT^ and NFIB^ΔIDR^ cells also showed differences at the gene expression levels. Approximately 130 genes were increased in NFIB^WT^ compared to the empty vector and most of them were unchanged in NFIB^ΔIDR^. Almost half of these genes had promoters or distal regulatory regions occupied by NFIB^WT^ and associated with higher ATAC-seq and H3K27ac signals (Extended Data Fig. 5i). GO analysis of upregulated genes revealed that overexpression of NFIB^WT^ but not NFIB^ΔIDR^ induced genes involved in cell differentiation, tissue development, cell locomotion and migration (Fig. 4f). Genes in these categories can be upregulated upon EWS-FLI1 depletion (Extended Data Fig. 5j) and are consistent with the increases in mesenchymal differentiation and migration observed after loss of the fusion protein^23,24^. In sum, our results show that the IDR of NFIB is necessary for robust binding to DNA, incorporation into DisP islands, activation of its full repertoire of target sites and regulation of downstream gene expression programs.

Given that an increasing number of IDRs found in TFs have been associated with phase separation and the formation of transcriptional condensates^10,13,46^, we considered whether the IDR of NFIB is capable of forming biomolecular condensates. We found that purified NFIB^IDR^ (a protein consisting of the NFIB IDR fused with enhanced green fluorescent protein, eGFP) formed liquid-like droplets that were decreased in the presence of higher salt concentrations (Extended Data Fig. 5k, top). In contrast, purified NFIB^ΔIDR^ (NFIB lacking the IDR domain and fused with eGFP) formed a few loose aggregates at low salt concentrations and no visible aggregates in higher salt conditions (Extended Data Fig. 5k, bottom). Similarly, the in vivo optoDroplet assay (which measures the ability of protein fragments fused to mCherry and the CRY2 photolyase domain to form liquid-like droplets upon light stimulation^47^) showed that NFIB^IDR^ readily forms droplets after 30 s of exposure to blue light (Fig. 4g). No droplets were observed for NFIB^ΔIDR^ under the same conditions. While these findings show that the IDR of NFIB is capable of mediating the formation of biomolecular condensates, the relative contributions of phase transitions and other mechanisms mediated by multivalent IDR interactions to endogenous NFIB function are yet to be determined^48^.

### NFIB is enriched in DisP islands in SCLCs

**Extended Data Fig. 6:**
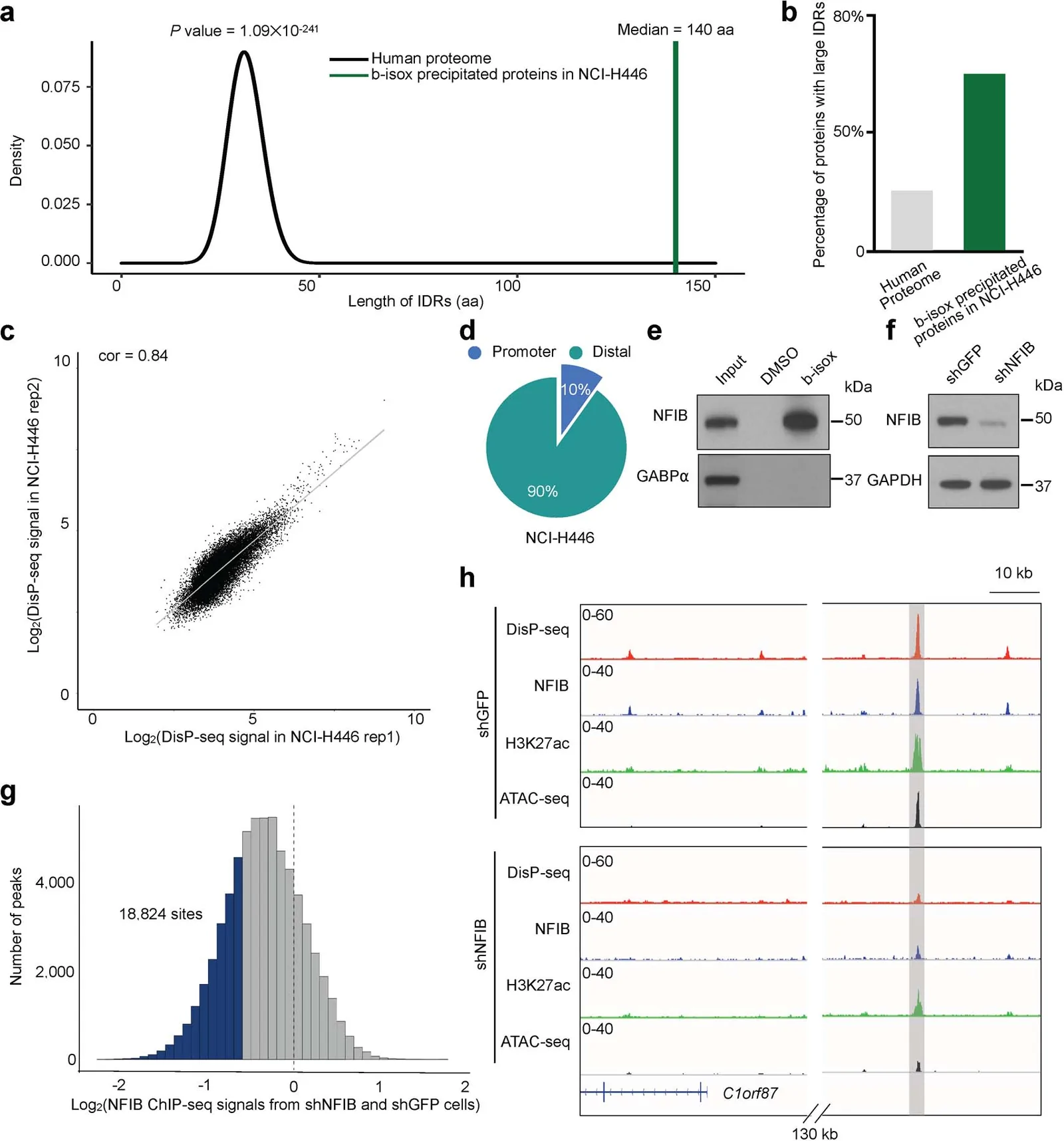
Analysis of DisP-seq signals in NCI-H446 cells. **a**, Graph showing the median length of IDRs found in b-isox precipitated nuclear proteins identified by mass spectrometry in NCI-H446 cells compared to permutations of equal numbers of proteins selected from the human proteome. The *P*-value was calculated by permutation test. **b**, Bar plot depicting the percentage of proteins with large IDRs (greater than 100 amino acids) in the human proteome and in b-isox precipitated nuclear proteins identified by mass spectrometry in NCI-H446 cells. **c**, Scatter plot showing DisP-seq signals for two biological replicates in NCI-H446 cells. The corresponding Pearson correlation coefficient is shown. **d**, Pie chart depicting the fraction of DisP-seq peaks with NFIB ChIP-seq signals in NCI-H446 cells. **e**, Western blot after b-isox precipitation shows that NFIB can be precipitated in NCI-H446 cells. **f**, Western blot of NFIB and GAPDH in NFIB depleted cells. GAPDH was chosen as a loading control. **g**, Histogram showing changes in NFIB ChIP-seq signals after depletion of NFIB (log_2_ fold scale). Peaks displaying more than a 1.5-fold decrease are shown in blue. **h**, Representative example of decreasing DisP-seq signals upon NFIB knockdown. DisP-seq, NFIB, H3K27ac ChIP-seq and ATAC-seq signals are shown at a region distal to the *C1orf87* gene. The distal region is highlighted in light gray.

Our initial DisP-seq experiments focused on Ewing sarcoma cells as a model where EWS-FLI1 provides a well-defined IDR-containing paradigm. We next sought to extend our findings to a different cellular context that is devoid of EWS-FLI1. For this purpose, we selected human SCLC NCI-H446 cell line, where NFIB has been shown to be highly expressed and can promote metastasis by enhancing chromatin accessibility at a large set of loci^41,49^. As in SKNMC cells, nuclear proteins detected by mass spectrometry after b-isox precipitation show higher median MobiDB IDR annotation length^27^ and a greater proportion of long IDRs compared to the human proteome (Extended Data Fig. 6a,b). DisP-seq in NCI-H446 cells and identified 19,516 peaks shared by two biological replicates (Extended Data Fig. 6c). Similar to our observations in Ewing cells, we found that about 92% of DisP-seq peaks were associated with distal regions. The remaining peaks were located at gene promoters in this SCLC model (Extended Data Fig. 6d).

**Fig. 5:**
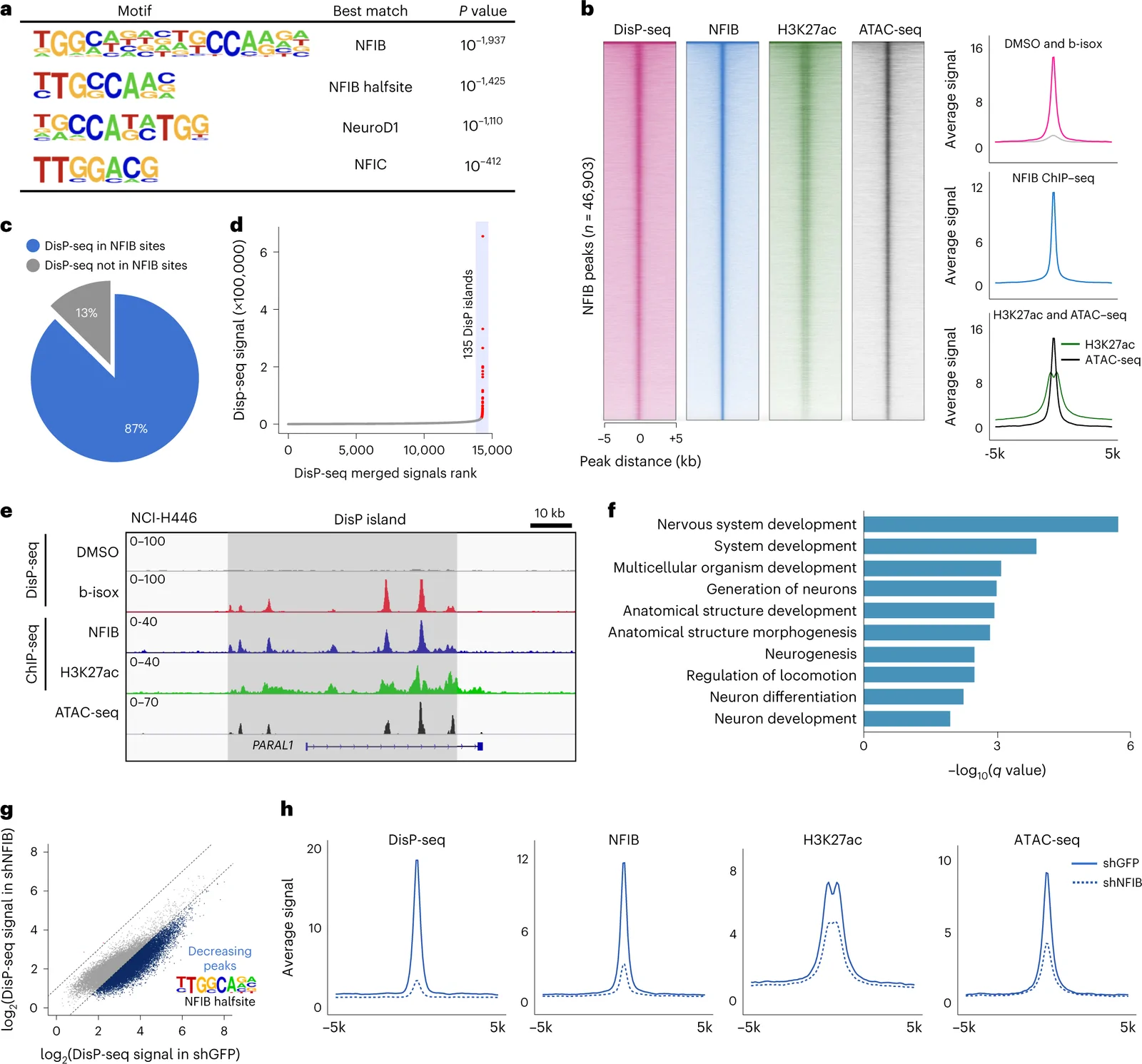
DisP-seq signals in SCLC NCI-H446 cells are associated with NFIB. **a**, Motif enrichment analysis for DisP-seq signals in NCI-H446 SCLC cells. The top four motifs identified are shown. **b**, Heatmaps (left) and composite plots (right) showing DisP-seq, NFIB, H3K27ac ChIP–seq and ATAC-seq signal densities for 46,903 NFIB peaks in NCI-H446 cells. For each heatmap, ±5 kb regions centered on the DisP-seq peaks are shown. **c**, Pie chart showing genome-wide distribution of all DisP-seq peaks in NCI-H446 cells. **d**, Identification of DisP islands in NCI-H446 cells. Distribution of merged DisP-seq signals. DisP islands are defined as the population of merged signals above the inflection point of the curve (slope = 1). DisP-seq signals within 20 kb were merged for this analysis. **e**, Representative example of DisP island with H3K27ac ChIP–seq and ATAC-seq signals (highlighted in light gray). **f**, GO analysis of genes associated with DisP islands in NCI-H446 cells. **g**, Scatter plot showing changes in DisP-seq peaks upon NFIB knockdown in NCI-H446 cells. 17,589 peaks displaying more than a twofold change are shown in blue (decreased). The top motif for decreasing peaks is shown on the right. **h**, Composite plots showing decreases in DisP-seq, NFIB and H3K27ac ChIP–seq and ATAC-seq upon NFIB depletion for 18,824 peaks with NFIB ChIP–seq signal decreases (>1.5-fold change).

Motif enrichment analysis showed that the top two motifs enriched at DisP-seq peaks corresponded to the NFIB full site and half site (Fig. 5a) and we verified these findings by confirming NFIB precipitation by b-isox in SCLC cells (Extended Data Fig. 6e) and performing ChIP–seq for endogenous NFIB. Remarkably, most DisP-seq peaks in NCI-H446 cells (87%) are associated with NFIB ChIP–seq signals (Fig. 5b,c). These locations were also positive for ATAC-seq and H3K27ac signals (Fig. 5b), indicating that they correspond to active regulatory elements. Because NFIB is associated with DisP islands in SKNMC cells, we sought to also explore the relationship between NFIB and DisP islands in SCLC cells. From the analysis of DisP-seq signals, we identified 135 DisP islands in NCI-H446 cells (Fig. 5d), all of which are associated with NFIB (Fig. 5e). NFIB can thus also be incorporated into DisP islands in SCLC cells. Potential target genes of DisP islands in this setting were enriched for GO annotations associated with neuronal function, including nervous system development, generation of neurons, neurogenesis, neuron differentiation and development (Fig. 5f).

We further tested the relationship between NFIB and DisP-seq signals by shRNA knockdown (Extended Data Fig. 6f). Fifty-one percent of DisP-seq peaks were downregulated after NFIB depletion and the top DNA motif for decreasing DisP-seq peaks corresponded to the NFIB half site (Fig. 5g). Sites with decreased NFIB signals were associated with robust decreases in DisP-seq, H3K27ac and ATAC-seq signals (Fig. 5h and Extended Data Fig. 6g,h). Taken together, our results in SKNMC and NCI-H446 cells show that DisP-seq can effectively detect TFs with prominent IDRs in various cellular contexts and that NFIB can be a major determinant of the DisP-seq landscape outside of Ewing sarcoma.

### Most DisP-seq peaks and DisP islands are cell-type-specific

**Extended Data Fig. 7:**
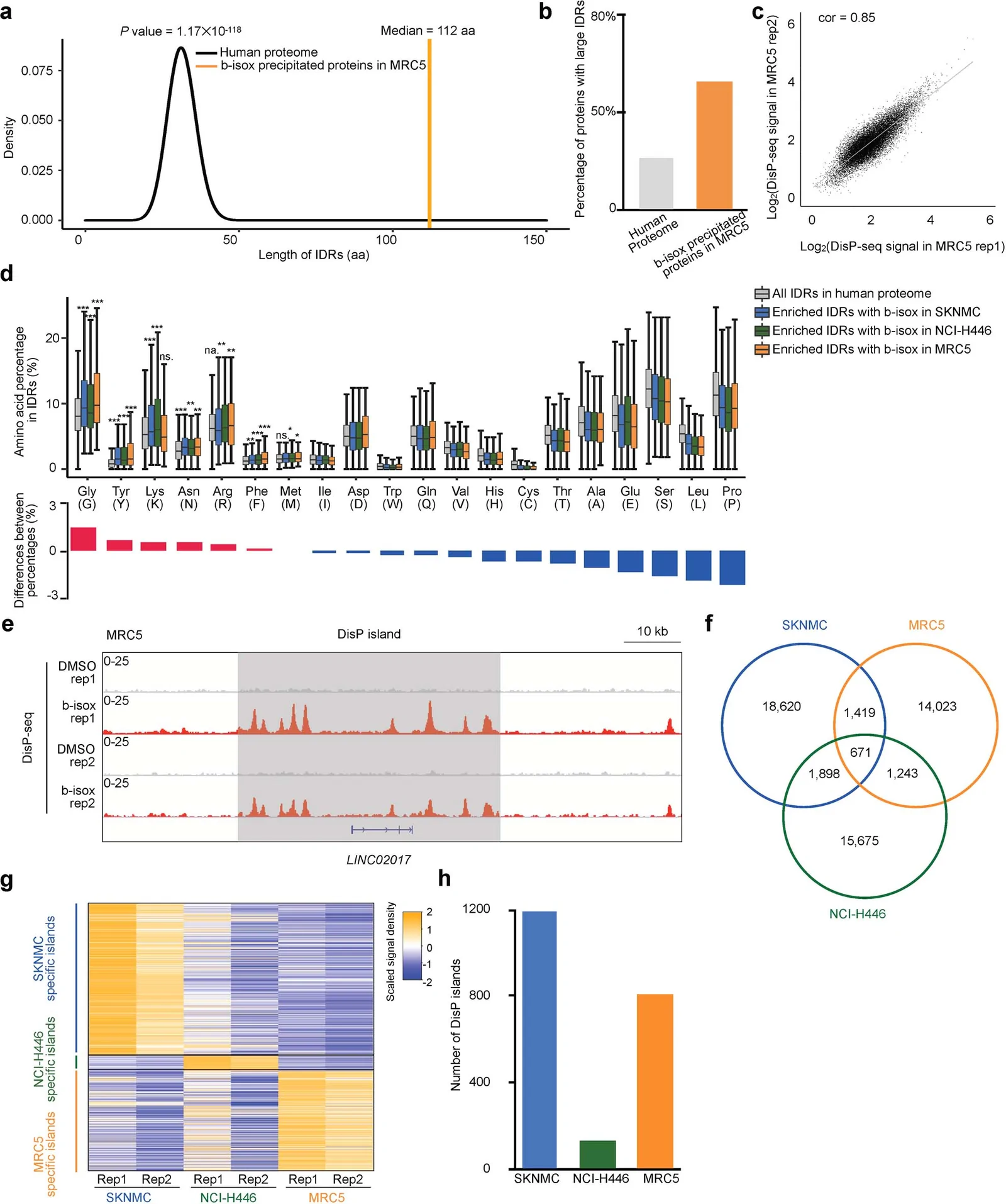
DisP-seq signals and DisP islands in different cell lines. **a**, Graph showing the median length of IDRs found in b-isox precipitated nuclear proteins identified by mass spectrometry in MRC5 cells compared to permutations of equal numbers of proteins selected from the human proteome. The *P*-value was calculated by permutation test. **b**, Bar plot depicting the percentage of proteins with large IDRs (greater than 100 amino acids) in the human proteome and in b-isox precipitated nuclear proteins identified by mass spectrometry in MRC5 cells. **c**, Scatter plot showing DisP-seq signals for two biological replicates in MRC5 cells. The corresponding Pearson correlation coefficient is shown. **d**, Top: Percent amino acid composition for IDRs found in b-isox precipitated nuclear proteins identified by mass spectrometry in each cell line compared with all IDRs in the human proteome (n = 2 biologically independent experiments). Bottom: Percent differences between the human proteome values and the average cell line values shown in the top graph. Median value is shown as a line within the boxplot, which spans from the 25th to 75th percentiles. Whiskers indicate a 1.5× inter-quartile range. **e**, Representative example of DisP island (highlighted in light gray). **f**, Venn diagram depicting the overlap among DisP-seq peaks in SKNMC, NCI-H446 and MRC5 cell lines. **g**, Heatmaps depicting DisP-seq signal intensities at unique DisP islands from each cell line. Two biological replicates are shown for each cell lines. **h**, Numbers of DisP islands in each cell line.

After analyzing DisP-seq peaks and DisP islands in two different tumor types (Figs. 1g and 5d), we next considered whether similar signals can be observed in a noncancer cell line model. For this purpose, we performed DisP-seq in the lung embryonic fibroblast cell line MRC5. As expected, analysis of nuclear proteins found by mass spectrometry in b-isox precipitates in MRC5 cells shows a similar pattern as other cell lines examined, with markedly longer median IDR lengths compared to the human proteome (Extended Data Fig. 7a–c). Having acquired mass spectrometry data for b-isox precipitates in three different cell lines, we also considered whether there is a systematic sequence bias for the IDRs in nuclear disordered proteins precipitated by b-isox. This analysis showed a modest enrichment for glycine, tyrosine and lysine residues (Extended Data Fig. 7d), suggesting that b-isox precipitation may have some selectivity for a subset of IDRs.

**Fig. 6:**
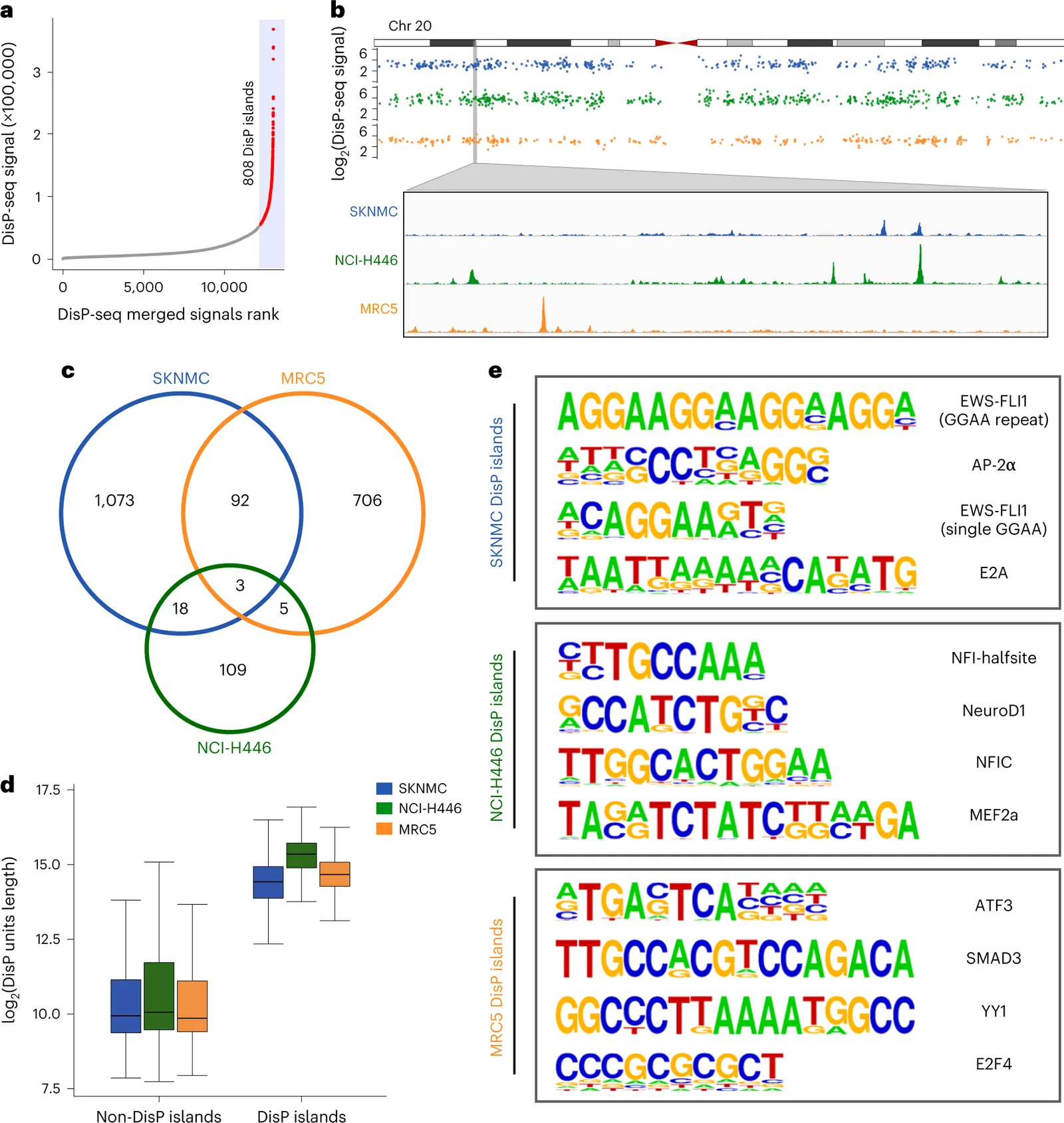
DisP islands in different cell lines. **a**, Identification of DisP islands in MRC5 cells. Distribution of merged DisP-seq signals. DisP islands are defined as the population of merged DisP-seq signals above the inflection point of the curve (slope = 1). DisP-seq signals within 20 kb were merged for this analysis. **b**, Top—distribution of DisP-seq signals on chromosome 20 in SKNMC, NCI-H446 and MRC5 cell lines. Bottom—zoom-in window from the top section. **c**, Venn diagram depicting the overlap among DisP islands in SKNMC, NCI-H446 and MRC5 cell lines. **d**, Box plots showing the length of DisP islands and DisP peaks in SKNMC, NCI-H446 and MRC5 cell lines (*n* = 2 biologically independent experiments). Median value is shown as a line within the boxplot, which spans from the 25th to 75th percentiles. Whiskers indicate a 1.5× interquartile range. **e**, Motif analysis for DisP islands in SKNMC, NCI-H446 and MRC5 cell lines. The top four motifs identified in each cell line are shown.

DisP-seq profiles for MRC5 cells showed a total of 808 shared DisP islands in two DisP-seq replicates in MRC5 cells (Fig. 6a and Extended Data Fig. 7e). We next compared single DisP-seq peaks and DisP islands from two cancer cell lines (SKNMC and NCI-H446) and MRC5 cells and found that most single DisP-seq peaks and DisP islands are cell-type-specific (Fig. 6b,c and Extended Data Fig. 7f,g). We also compared the length and numbers of DisP islands in SKNMC, NCI-H446 and MRC5 cells. Interestingly, NCI-H446 cells have fewer islands but these are substantially longer (Fig. 6d and Extended Data Fig. 7h), suggesting that the presence of high levels of NFIB may promote more extensive clustering.

Finally, we performed DNA motif enrichment analysis to identify disordered TFs associated with these different sets of DisP islands (Fig. 6e). This analysis showed that the top motifs in DisP islands are associated with distinct sets of TFs in SKNMC cells (AP-2α, EWS-FLI1 and E2A), NCI-H446 cells (NFI family, NeuroD1 and MEF2a) and MRC5 cells (ATF3, SMAD3, YY1 and E2F4). Notably, these TFs contain large disordered domains and include the TF YY1, which has previously been implicated in the formation of biomolecular condensates^50^. Together, these results show that DisP-seq peaks and DisP islands are distinctive features of the genomic landscape in different cell types that can be profiled systematically in these different contexts.

## Discussion

We show that DisP-seq is a method that can generate maps of the genome-wide distribution of DNA-associated disordered proteins by detecting these proteins simultaneously in an antibody-independent manner. Our results in several cell types examined reveal that DNA-associated disordered proteins are distributed widely in the genome and are arranged in peaks and large clusters (DisP islands) associated with different types of chromatin states and regulatory elements. Furthermore, examination of gene regulation in cancer cells shows that the cell-type specific organization patterns of DNA-associated disordered proteins can have important roles in pathologic and physiologic gene regulation mechanisms. Given that DisP-seq is antibody-independent, it can also lead to the identification of disordered TFs that have critical roles in IDR-dependent regulatory programs. DisP-seq can thus be widely applied to uncover IDR-dependent mechanisms and effectors in many biological processes and disease states without prior knowledge.

Comparing ChromHMM results and DisP-seq signals in SKNMC cells revealed that most DisP-seq peaks were associated with enhancers (Fig. 1d–f). This finding is consistent with previous studies showing that IDR-containing TFs and cofactors can form transcriptional condensates at enhancer regions^6,13^. Interestingly, DisP-seq peaks are associated with a larger number of weak enhancers than active enhancers. This shows that disordered proteins may also accumulate at enhancers in the absence of activation marks and may reflect the presence of repressors or of a chromatin state poised for future activation. Given that DisP-seq can in principle detect proteins that bind DNA directly (such as TFs and histones) or are indirectly associated with DNA (such as transcriptional cofactors and chromatin regulators), both categories of proteins may contribute to the signals observed at enhancers. While this study focuses on the identification of TFs using motif searches, other types of analysis may reveal the contributions of other DNA-associated proteins to DisP-seq profiles.

While DisP-seq peaks are most frequently detected at enhancers, it is notable that they are only found in subsets of these regulatory elements. Similarly, DisP-seq peaks overlap only a minority of open sites defined by ATAC-seq and can also be present at sites devoid of ATAC-seq signals. These results show that the IDR content of similar regulatory elements can be very heterogenous and, together with the cell type specificity of DisP-seq signals, supports the value of in-depth profiling of DNA-associated disordered proteins in different cellular states. Our profiles also show significant differences in DisP-seq signals for TFs as exemplified by the strong signals observed for AP-2α and NFIB and the low levels observed at sites occupied by the ETS factor GABPα. In this light, it will be interesting to consider whether the relative strength of signals obtained by b-isox or other chemical precipitation methods may provide a means of classifying TFs and other DNA-associated proteins into different functional classes. It is also worth noting that, as demonstrated by the modest enrichment for certain amino acids in our mass spectrometry data, b-isox may have some selectivity for a subset of disordered proteins that may be linked to particular functional properties.

Our results show that large DisP-seq clusters, which we named DisP islands, are a prominent finding in genome-wide landscapes of DNA-associated disordered proteins. These clusters represent high local concentrations of disordered proteins and we find that they can occur as part of pathologic and physiologic gene regulation programs. TFs with IDRs, including EWS-FLI1, have been shown to participate in the formation of biomolecular condensates and to activate transcription in association with the recruitment of RNA polymerase II^46^. Our data further show that DisP islands exhibit features consistent with large-scale cooperative activity as has been proposed for the assembly of transcriptional condensates in regulatory elements^51^. For example, we observe increased signals for DisP-seq and histone modifications at peaks inside DisP islands compared to those outside of these locations. Moreover, we also directly observed that binding of EWS-FLI1 and NFIB in DisP islands is coordinated over distances beyond direct cooperative binding or histone competition mechanisms, which typically occur within 50 bp^39^. Together with our finding that the NFIB IDR can mediate condensate formation and is required for incorporation into DisP islands, these results suggest that clusters of DisP-seq peaks may be engaged in the formation of co-condensates containing different disordered proteins. Because DisP islands can occur in both active and inactive chromatin environments, the clustering of disordered proteins may explain the coordinate binding of TFs, even in the absence of strong activation signals.

Our DisP-seq profiles of Ewing sarcoma cells after EWS-FLI1 knockdown show that changes in cellular states can lead to large-scale reorganization of the DisP island landscape (Fig. 3k). Remarkably, we also find that gains and losses in DisP islands are connected through the disordered TF NFIB, which is initially sequestered at EWS-FLI1 DisP islands and relocates to activate mesenchymal differentiation. This IDR-dependent sequestration affects the selection of NFIB binding to DNA. Thus, in addition to the well-known activation of enhancers and target genes by EWS-FLI1 (refs. ^35,36^), pathologic EWS-FLI1 DisP islands can also suppress differentiation in tumor cells through sequestration of other disordered TFs. These findings raise the possibility that similar mechanisms may be operative in DisP islands observed in a variety of settings, including other tumors driven by EWS fusion proteins.

Together, our methodology and analyses in multiple cell lines show that DNA-associated disordered proteins are distributed across different chromatin states and can form large functional clusters that promote coordinated DNA-binding and regulatory activities. We thus expect that the broad application of DisP-seq for in-depth analysis of cellular states will provide a path toward understanding the important relationship between the organization of DNA-associated disordered proteins and gene regulation programs in biology and disease.

## Methods

### Cell lines

Cell lines were obtained from ATCC. SKNMC Ewing sarcoma cells were grown in RPMI-1640 (Gibco, 11875). HEK293T was grown in DMEM (Gibco, 11995). MRC5 was grown in EMEM (ATCC, 30-2003) and NCI-H446 SCLC cell line was grown in specific RPMI-1640 Medium (Gibco, A1049101). All media were supplemented with 10% FBS and cells were cultured at 37 °C with 5% CO_2_. Cells were maintained and split every 2–4 d according to ATCC recommendations.

### Lentiviral generation

Lentivirus was produced in 293T Lenti-X cells (Takara, 632180) by LT1 Transfection Reagent (Mirus Bio, MIR 2305) transfection with gene delivery vector and packaging vectors pCMV-VSV-G (Addgene, 8454) and pCMV-dR8.2 dvpr (Addgene, 8455) plasmids. Viral supernatants were collected 48 h and 72 h after transfection and concentrated using Lenti-X concentrator (Takara, 631232). Virus-containing pellets were resuspended in PBS and added dropwise on cells in presence of media supplemented with 6 mg ml^−1^ polybrene. Selection of lentivirally-infected cells was achieved with puromycin used at 2 μg ml^−1^ for 7 days. Overexpression or knockdown efficiency was determined by western blot analysis.

### Transient transfections

HEK293T cells were plated and grown to 60% confluency before transfection using LT1 Transfection Reagent (Mirus Bio, MIR 2305) according to the manufacturer recommendations and were collected after 48 h.

### Plasmid construction

All shRNAs were in the pLKO.1 backbone and the sequences of shRNAs are listed in Extended Data Table 1. To construct expression plasmids for NFIB^WT^, the full-length NFIB was amplified from NCI-H446 cDNAs and inserted into pENTR3C Dual Selection Vector (Thermo Fisher Scientific, A10464) with NotI-HF (NEB, R3189S) and XbaI (NEB, R0145S).

NFIB^ΔIDR^ was generated using the Q5 Site-Directed Mutagenesis Kit (NEB, E0554S). Plasmids for protein purification were constructed by amplifying and inserting NFIB^IDR^ and NFIB^ΔIDR^ into the His-MBP-eGFP vector using HiFi DNA Assembly Cloning Kit (NEB, E5520S). For OptoDroplets assay, NFIB^IDR^ and NFIB^ΔIDR^ were amplified and inserted into Cry2-mCh-NLS vector using HiFi DNA Assembly Cloning Kit (NEB, E5520S).

### Western blot analysis

Western blotting was performed using standard protocols. Primary antibodies (1:1,000 for anti-FLI1, anti-AP-2α, anti-NFIB, anti-V5, anti-HA and anti-GABPα; 1:10,000 for anti-GAPDH) used for western blotting are listed in Extended Data Table 1. Secondary antibodies were goat antirabbit and goat antimouse immunoglobulin G-horseradish peroxidase-conjugated (Bio-Rad, 1:10,000 dilution). Membranes were developed using Western Lightning Plus-ECL enhanced chemiluminescence substrate (PerkinElmer, NEL104001EA) and visualized using photographic films.

### Protein sequence analysis

Protein sequences from the Uniprot database were analyzed using VSL2 algorithm from Predictor of Natural Disordered Region (PONDR, http://www.pondr.com/)^31^and Metapredict V2 online^32^.

### Immunoprecipitation

For each sample, 5 million collected cells were resuspended in 500 μl IPH buffer (50 mM Tris–HCl pH 8, 150 mM NaCl, 5 mM EDTA, 0.5% NP-40 and 10% glycerol supplemented with 1× Protease/Phosphatase inhibitors (Thermo Fisher Scientific, 78444), 0.1 mM PMSF) before sonication in a QSONICA 800R instrument (30 s on and 30 s off, 15 min in total, 4 °C). Protein supernatant was then collected after centrifugation for 15 min at 18,400*g* and 4 °C. The proteins were incubated overnight at 4 °C with 2 μg of the indicated antibodies (listed in Extended Data Table 1) in the presence of protein G Dynabeads (Life Technologies, 10004D) and 100 mg ml^−1^ Ethidium Bromide (Invitrogen, 15585-011). Beads were washed five times with IPH buffer and eluted by boiling in 2× Laemmli buffer (Boston BioProducts, BP-111R).

### Biotinylated isoxazole-mediated precipitation and mass spectrometry

These assays were performed as previously described^20^ with slight modifications. b-isox (Sigma-Aldrich, 900572-1MG) was reconstituted in DMSO. Briefly, 10 million cells were resuspended in 1 ml lysis buffer (20 mM Tris–HCl pH 7.4, 150 mM NaCl, 5 mM MgCl_2_, 0.5% NP-40 and 10% glycerol supplemented with 1× Protease/Phosphatase inhibitors (Thermo Fisher Scientific, 78444), 0.1 mM PMSF and 20 mM beta-mercaptoethanol) and incubated for 30 min with rotation at 4 °C. The supernatant was then collected after centrifugation for 15 min (18,400*g*, 4 °C). A 10% whole-cell extract control was collected and the remaining proteins were divided into two aliquots before the addition of DMSO and 100 μM b-isox, respectively. The reaction solutions were incubated at 4 °C for 1 h with rotation and centrifuged for 15 min (18,400*g*, 4 °C). The supernatant was removed and pellets were washed twice in supplemented lysis buffer and then resuspended in 2× Laemmli buffer (Boston BioProducts, BP-111R). The samples were analyzed with 4–12% Tris-Glycine gradient gels (Invitrogen, NW04120BOX), and western blotting was performed using standard protocols.

For mass spectrometry, samples were run on a 4–12% Tris-Glycine gradient gel (Invitrogen, NW04120BOX) and subjected to Coomassie staining. Total bands were then cut for each sample and submitted to the Taplin Biological Mass Spectrometry Facility (Harvard Medical School) for analysis.

### DisP-seq

We used 10 million cells for DMSO control and b-isox samples. Cells were trypsinized and washed with cold PBS. The nucleus of cells was isolated with Nuclei EZ Prep Kit (Sigma-Aldrich, NUC101-1KT) following the manufacturer’s instructions. Isolated nuclei were resuspended in 200 μl prewarmed MNase reaction buffer (50 mM Tris–HCl pH 7.4, 320 mM sucrose, 4 mM MgCl_2_, 1 mM CaCl_2_ supplemented with 1× Protease/Phosphatase inhibitors (Thermo Fisher Scientific, 78444) and 0.1 mM PMSF) and digested by 6 U MNase (Thermo Fisher Scientific, EN0181) for 1 min at 37 °C. Then 800 μl b-isox lysis buffer (20 mM Tris–HCl pH 7.4, 187.5 mM NaCl, 5 mM MgCl_2_, 0.625% NP-40 and 12.5% glycerol supplemented with 1× Protease/Phosphatase inhibitors (Thermo Fisher Scientific, 78444), 0.1 mM PMSF and 25 mM beta-mercaptoethanol) was added to quench the digestion. The digested nuclei were incubated for 30 min with rotation at 4 °C. The supernatant was then collected after centrifugation for 15 min (18,400*g*, 4 °C). Ten percent of samples were saved as Input and the remaining samples were divided into two aliquots before the addition of DMSO and 100 μM b-isox, respectively. The reaction solutions were incubated at 4 °C for 1 h with rotation and centrifuged for 15 min (18,400*g*, 4 °C). The supernatant was removed and pellets were washed twice in wash buffer (20 mM Tris–HCl pH 7.4, 150 mM NaCl, 5 mM MgCl_2_, 0.5% NP-40, 10% glycerol supplemented with 1× Protease/Phosphatase inhibitors (Thermo Fisher Scientific, 78444), 0.1 mM PMSF and 20 mM beta-mercaptoethanol). Next Input and pellets were resuspended in 200 μl elution buffer (10 mM Tris–HCl pH8, 0.1 % SDS, 150 mM NaCl and 5 mM DTT) by shaking (600 rpm) at 65 °C for 1 h. After that, samples were treated with 2 μl RNase (Roche, 43813100) at 37 °C for 30 min and then with 6 μl proteinase K (Invitrogen, 25530049) by shaking (600 rpm) at 65 °C for 3 h. DNAs were extracted with AMP Pure beads (Beckman Coulter, A63881) and eluted with 40 μl 10 mM Tris–HCl pH 8.0. Then eluted DNAs were quantified with Qubit dsDNA HS Assay kit (Invitrogen, Q32854) and 2 ng DNAs were used to prepare sequencing libraries with Ultralow V2 DNA-Seq Library Preparation Kit (NuGEN, 0344NB-A01) and were sequenced with the Nextseq 500 Illumina genome analyzer.

### ChIP–seq

ChIP assays were carried out on 5 million cells per sample, following the procedures described previously^52^. In brief, chromatin from formaldehyde-fixed cells was fragmented to a size range of 200–700 bases with a Branson 250 Sonifier. Solubilized chromatin was immunoprecipitated with 5 μg antibodies against AP-2α (Santa Cruz, sc-12726X), NFIB (Sigma-Aldrich, HPA003956), H3K27ac (Active Motif, 39133), V5 (Cell Signaling, 13202) and FLI1 (Abcam, ab15289) at 4 °C overnight. Antibody–chromatin complexes were pulled down with protein G Dynabeads (Life Technologies, 10004D), washed, and then eluted. After cross-link reversal and RNase (Roche, 43813100) and proteinase K (Invitrogen, 25530049) treatment, immunoprecipitated DNA was extracted with AMP Pure beads (Beckman Coulter, A63881). ChIP DNA was quantified with Qubit dsDNA HS Assay kit (Invitrogen, Q32854). ChIP DNA samples were used to prepare sequencing libraries with Ultralow V2 DNA-Seq Library Preparation Kit (NuGEN, 0344NB-A01) and DNA samples were sequenced with the Nextseq 500 Illumina genome analyzer.

### ATAC-seq

ATAC-seq analysis was performed as recently described with some modifications^53,54^. Briefly, 5 × 10^4^ cells were pretreated with 200 U ml^−1^ DNase (Worthington, LS002006) in the culture medium for 30 min at 37 °C, then washed with PBS twice. Cell pellets were resuspended in 50 μl freezing media (10% DMSO, 50% FBS and 40% complete media) and transferred in an isopropyl alcohol chamber at −80 °C overnight. The next day, the frozen cell pellets were thawed and first incubated in L1 buffer (10 mM Tris–HCl pH 7.4, 10 mM NaCl, 3 mM MgCl_2_, 0.1% Digitonin, 0.1% Tween-20 and 0.1% NP-40 supplemented with 1× Protease/Phosphatase inhibitors (Pierce)) for 3 min then resuspended in L2 buffer (10 mM Tris–HCl pH 7.4, 10 mM NaCl, 3 mM MgCl_2_ and 0.1% Tween-20 supplemented with 1× Protease/Phosphatase inhibitors (Thermo Fisher Scientific, 78444)), centrifugated and resuspended in tagmentation buffer (25 μl 2× TD buffer (Illumina, 15027865), 2.5 μl Tn5 transposase (Illumina, 15027866), 16.5 μl PBS, 0.5 μl 1% digitonin, 0.5 μl 10% Tween-20, and 5 μl water) for additional 30 min at 37 °C, following manufacturer recommendations (Nextera DNA Sample Prep Kit, Illumina, 20015882). After DNA purification, adapter sequences were added to the fragmented DNA by PCR. Purified PCR products were sequenced using the Nextseq 500 Illumina genome analyzer.

### RNA-seq

Total RNAs were isolated from cells using the NucleoSpin RNA Plus kit (Takara, 740984.50) and 500 ng RNAs were used to prepare sequencing libraries with CORALL Total RNA-Seq Library Prep Kit (LEXOGEN, 146) and were sequenced with the Nextseq 500 Illumina genome analyzer.

### Protein expression and purification

Expression plasmids with His tag were individually transformed into an *Escherichia coli* expression strain BL21 (NEB, C2527H). After transformation, a single colony was incubated in 5 ml terrific broth (TB) media (Sigma-Aldrich, T0918-1KG) supplemented with 100 μg l^−1^ kanamycin at 250 rpm, 37 °C. After overnight growth, the culture was diluted 250-fold into 100 ml TB medium supplemented with 100 μg l^−1^ kanamycin. Absorbance was monitored at a wavelength of 600 nm, and upon reaching an optical density (OD600) of 0.6, IPTG (Roche, 10724815001) was added to TB medium at the concentration of 0.5 mM for the induction of protein expression. After overnight incubation at 200 rpm, 16 °C, cell pellets were collected by centrifugation (1,500*g*, 10 min, 4 °C), and then pellets were frozen at −80 °C overnight. For protein purification, pellets were resuspended in 20 ml lysis buffer (50 mM Tris pH 7.5, 1 M NaCl, 10 mM imidazole, 0.5 mM PMSF) with 1 mg ml^−1^ lysozyme (Sigma-Aldrich, 62970-1G-F) rotated at 4 °C for 30 min, and sonicated by QSONICA Q700 sonicator (15% amplitude, 10 s on, 20 s off, 2 min 2×) at 4 °C. After centrifugation at 18,400*g* for 10 min at 4 °C, the supernatant cell lysates were filtered through a 0.45 μm filter and then loaded onto a Chromatography Column (Bio-Rad, 7321010) with 2 ml Ni Sepharose (GE Healthcare, 17-5318-01), which was pre-equilibrated in wash buffer (50 mM Tris pH 7.5, 1 M NaCl, 20 mM imidazole and 0.5 mM PMSF) at 4 °C. The loaded column was washed with 20 column volumes (CV) of wash buffer at 4 °C. Proteins were eluted in 3 CV of elution buffer (50 mM Tris pH 7.5, 1 M NaCl, 250 mM imidazole and 0.5 mM PMSF), and then concentrated using Amicon Ultra-15 Centrifugal Filter (Millipore, UFC901008) by spinning at 4,000*g* for 30 min at 4 °C. The concentrated proteins were dialyzed in 500 ml dialysis buffer (20 mM Tris pH 7.5, 500 mM NaCl, 2 mM DTT, 20% Glycerol and 0.1 mM PMSF) at 4 °C overnight and then stored at −80 °C.

### In vitro droplet formation assay

The in vitro droplet formation assay was performed as described previously^55^. The purified proteins were assembled by diluting the protein from a high salt-containing storage buffer into droplet buffer (20 mM Tris pH7.5, 150 mM NaCl or 300 mM NaCl and 4% polyethylene glycol). Samples were prepared on a 12-well multiwell glass bottom culture plate (MatTek, P12G-1.5-14-F) and were imaged within 30 min after drop assembly with a Zeiss LSM 710 Confocal equipped with a 63 × 1.40 oil objective.

### optoDroplet assay

The assay was performed as described previously^47^. The cells were transfected with 200 ng of plasmid encoding CRY2-mCherry constructs. The cell culture medium was changed after 48 h post-transfection, and cells were visualized on a Zeiss LSM 710 confocal microscope equipped with an incubation chamber and a heated stage at 37 °C. Droplet formation was induced using scans with the 488 nm laser for 30 s. The images were acquired with a 63 × 1.40 oil objective.

### DisP-seq analysis

The DisP-seq and all the other sequencing data were converted to fastq files by Illumina Casava v2.19. We used DISPbind (v.1.0.2) for DisP-seq data processing with default settings. In brief, DisP-seq paired sequenced reads were aligned to hg19 genome using bwa v.0.7.12 (ref. ^56^) with default settings. After removal of duplicate reads using picard-tools v.1.95 (https://broadinstitute.github.io/picard/), reproduced precipitated DNA fragments by filling the gap region between paired reads. The fragments were normalized to 10 M reads to generate density maps. IGV^57^ was used for visualization of DisP-seq signals. MACS2 (ref. ^58^) v. 2.2.7.1 was used for peak calling with parameter: --nomodel -B --SPMR -f BAMPE --broad. Peaks with *q* value less than 10^−5^ were used for further analysis. DisP-seq map density signals were quantitated using python (pyBigWig v0.3.18) as the average read counts at locus of 1 kb window.

To compare DisP-seq signal changes between shGFP and shEWS-FLI1 (or shNFIB), we combined the DisP-seq peaks from both samples and used the union for DisP-seq signal calculation. Then, we calculated the average signal between replicates. Up- and down-regulated DisP-seq peaks were defined as changes greater than twofold between shEWS-FLI1 (or shNFIB) and shGFP.

### DisP islands analysis

We used DISPbind (v.1.0.2) for DisP island identification with default settings. Briefly, we grouped DisP-seq peaks within 20 kb into merged DisP regions and ranked these regions by total DisP-seq signals. Rank and signals were scaled (range 0–1) and DisP islands were defined as regions beyond the point with a tangent slope of 1. The comparison between DisP islands and super enhancers was performed using bedtools by downloading the super enhancer annotation for SKNMC cells from SEdb (http://www.licpathway.net/sedb/index.php)^59^. Differences in DisP islands were calculated by overlapping DisP islands in shGFP and shEWS-FLI1 SKNMC cells and selecting DisP islands that are specific for each condition. We defined shGFP-specific islands as ‘lost DisP islands’ and shEWS-FLI1–specific islands as ‘gained DisP islands’. We further subdivided lost DisP islands into pattern A (272 islands with EWS-FLI1 binding) and pattern B (214 islands without EWS-FLI1 binding). Similarly, new DisP islands were subdivided according to whether they contained an NFIB peak with more than twofold ChIP–seq signal increase as follows: pattern C (1,070 islands with increased NFIB ChIP–seq signals) and pattern D (236 DisP islands without increased NFIB ChIP–seq signals). Genes associated with gained DisP islands were identified within ±100 kb genomic regions of DisP islands in SKNMC shFLI1 cells. Upregulated genes (fold ≥ 2 and RPKM ≥ 5 in shEWS-FLI1) were selected for GO analysis.

### ChIP–seq processing

ChIP–seq sequencing results were aligned to the hg19 genome using bwa v.0.7.12 with default settings. After the removal of duplicate reads using picard-tools v.1.95, we extended aligned reads to 200 bp to approximate fragment sizes. And the density maps were normalized to 10 M reads. IGV was used to visualize ChIP–seq coverage maps. ChIP–seq peaks were identified with MACS2 v.2.2.7.1 with a *q*-value of 10^−5^. The narrow peak setting was used for TFs while broad peaks were called for histone markers. Peaks within 2 kb of TSS were considered promoter sites and the remaining sites were considered distal sites. Chromatin and TF signals associated with peaks were quantified using python (pyBigWig) as the average read counts in 1 kb windows.

### ATAC-seq processing

ATAC-seq reads were aligned to the hg19 genome using bwa v.0.7.12 with default settings. Reads that aligned in the proper orientation and on the same chromosome were then filtered to exclude PCR duplicates and processed as previously described^60^. We normalized the density maps to 10 M reads and visualized the results by IGV. ATAC-seq peaks were identified with MACS2 v.2.2.7.1 with parameter:--nomodel -B --SPMR -f BAMPE. Peaks passing a *q*-value cutoff 10^−5^ were kept for further analysis.

### Mass spectrometry data analysis

The intensity values and sum intensity of mass spectrometry data were determined by using the retention time and the *m*/*z* value to search for the peak height of each peptide in the raw data (GFY Core Version 3.8). Proteins identified from both replicates were used for analysis. Subcellular localization data from Uniprot (https://www.uniprot.org/)^61^, was used to select nuclear proteins for further analysis. The disorder annotation ‘prediction-disorder-th_50’ from MobiDB (Version 4.1.0)^27^ was used to calculate the length of IDRs. The proteins with a total length of IDRs greater than 100 were defined as having large IDRs. For permutation testing, 3,000 random samples of the same number of proteins were selected from the human proteome. To analyze the amino acid composition for b-isox enriched IDRs, we extracted the IDRs of b-isox enriched nuclear proteins from MobiDB and used IDRs of the human proteome as controls. ProtParam was used for the amino acid composition analysis^62^.

### Heatmap visualization

Signal of DisP-seq, ChIP–seq and ATAC-seq samples were computed by bwtool (version 1.0)^63^ with the following parameters 5,000:5,000 -tiled-averages = 100. Signal density matrices were plotted as heatmap by R package gplots.

### A/B compartment distribution of DisP-seq

The ENCODE SKNMC Hi-C A/B compartment density map was downloaded from Gene Expression Omnibus (GEO) series: GSE105914. A/B regions were used for the DisP-seq A/B compartment distribution analysis by overlapping DisP-seq peaks in SKNMC cells.

### Chromatin state analysis

Histone modification profiles for SKNMC cells (H3K4me3, H3K27ac, H3K4me1 and H3K27me3 ChIP–seq datasets) were downloaded from GEO series: GSE61953 using prefetch (v.2.8.0). We used ChromHMM^28^ v.1.22 to define chromatin states in SKNMC cells based on ChIP–seq data for histone modifications (H3K4me3, H3K27ac, H3K4me1, H3K9me3 and H3K27me3). We used 6 states for further analyses because this captured all the major combinations of chromatin marks. To annotate the chromatin states of DisP-seq peaks, we overlapped DisP-seq peaks with 6 states defined by ChromHMM using bedtools in SKNMC cells. The dominant overlapped state was assigned as the chromatin state for each DisP-seq peak. To evaluate the chromatin state of DisP-seq signals, we overlapped SKNMC DisP-seq peaks with ChromHMM-defined genomic regions by OLOGRAM v1.6.2 (ref. ^64^) with parameter: gtftk ologram -ms 40 -mn 10 -z -c hg19 -V 3 --force-chrom-peak --force-chrom-more-bed.

### RNA-seq processing and analysis

SKNMC RNA-seq samples for EWS-FLI1 knockdown experiments were downloaded from GEO series GSE61953 using prefetch (v.2.8.0). Reads were aligned using STAR v.2.4.0h (ref. ^58^). Aligned fragments were quantified using featureCounts^65^, and FPKM expression values were calculated for hg19 RefSeq genes. We used DEseq2 v3.10 (ref. ^66^) to perform the differential expression analysis for NFIB expression studies. Genes with 1.5-fold changes and *P* value < 0.05 were defined as differentially expressed genes. GO analysis was performed using GSEA website (https://www.gsea-msigdb.org/gsea/index.jsp).

### Motif analysis

HOMER v.4.7 was used for motif analysis. The motifs for DisP-seq were identified by findMotifsGenome.pl in HOMER^67^ with the following parameters: -size given -len 4,5,6,7,8,9,10,12,16. ‘annotatePeaks.pl’ was used for annotation of peaks with selected motifs.

### Statistics and reproducibility

All DisP-seq, RNA-seq, western blot and imaging experiments were repeated in biological duplicate with similar results.

### Statistics

*P* values for binding motifs were calculated using HOMER. The *P* values were calculated using two-sided *t*-tests. The GO analysis q-values were calculated from hypergeometric *P* values after correction for multiple hypothesis testing according to the Benjamini and Hochberg method. Pearson correlation coefficient values were calculated between DisP-seq replicates. *P* values for overlaps between SKNMC DisP-seq peaks and different chromatin state regions were calculated by OLOGRAM^64^.

### Reporting summary

Further information on research design is available in the Nature Portfolio Reporting Summary linked to this article.

## Online content

Any methods, additional references, Nature Portfolio reporting summaries, source data, extended data, supplementary information, acknowledgements, peer review information; details of author contributions and competing interests; and statements of data and code availability are available at https://doi.org/10.1038/s41587-023-01737-4.

## Supplementary information


Reporting Summary


## Source data


Source Data Fig. 2Unprocessed western blots.
Source Data Extended Data Table 1Mass spectrometry data for b-isox precipitation.


## Figures and Tables

**Extended Data Table 1:**
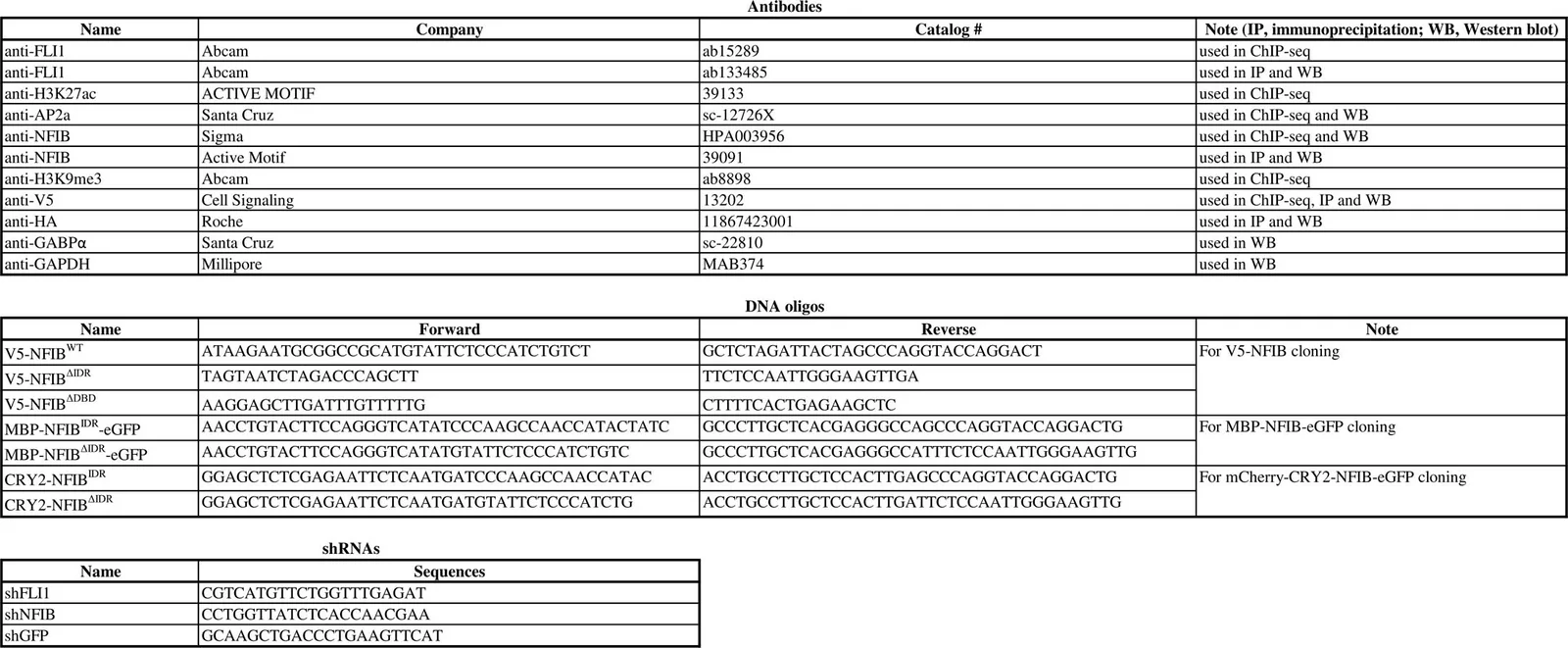
Antibodies, primers and shRNAs used in this study

Source data

**Extended Data Table 2:**
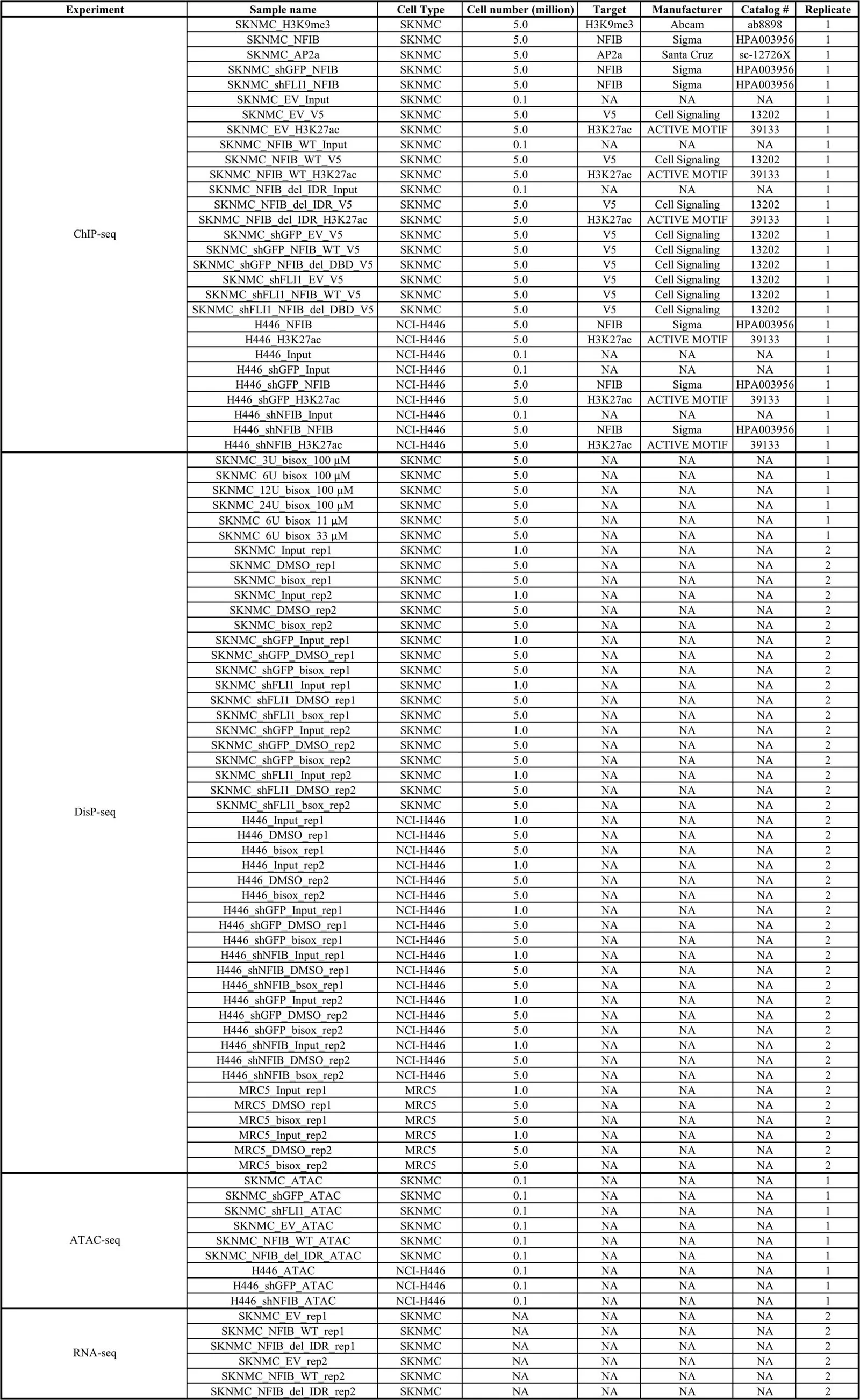
Information for sequencing files used in this study

## Data Availability

All next-generation sequencing datasets including those of DisP-seq, ChIP–seq, ATAC-seq and RNA-seq generated for this study are deposited in the NCBI GEO under the accession number GSE190963 (ref. ^68^). Source data are provided with this paper.

## Code availability

DISPbind for DisP-seq data processing and analysis can be accessed at https://github.com/rdong08/DISPbind (ref. ^69^).
