# Supplementary material for: DisP-seq reveals the genome-wide functional organization of DNA-associated disordered proteins
Source: Nat Biotechnol. 2023 Apr 10;42(1):52–64. doi: 10.1038/s41587-023-01737-4 (PMC10791585; doi:10.1038/s41587-023-01737-4)
Supplement: Supplementary file 1 — Reporting Summary [file 41587_2023_1737_MOESM1_ESM.pdf]

nature portfolio

Corresponding author(s): Miguel N. Rivera

Last updated by author(s): 2023-2-28

Reporting Summary

Nature Portfolio wishes to improve the reproducibility of the work that we publish. This form provides structure for consistency and transparency in reporting. For further information on Nature Portfolio policies, see our Editorial Policies and the Editorial Policy Checklist.

Statistics

For all statistical analyses, confirm that the following items are present in the figure legend, table legend, main text, or Methods section.

n/a	Confirmed	
☐	☒	The exact sample size (*n*) for each experimental group/condition, given as a discrete number and unit of measurement
☐	☒	A statement on whether measurements were taken from distinct samples or whether the same sample was measured repeatedly
☐	☒	The statistical test(s) used AND whether they are one- or two-sided<br>*Only common tests should be described solely by name; describe more complex techniques in the Methods section.*
☒	☐	A description of all covariates tested
☒	☐	A description of any assumptions or corrections, such as tests of normality and adjustment for multiple comparisons
☐	☒	A full description of the statistical parameters including central tendency (e.g. means) or other basic estimates (e.g. regression coefficient) AND variation (e.g. standard deviation) or associated estimates of uncertainty (e.g. confidence intervals)
☐	☒	For null hypothesis testing, the test statistic (e.g. *F*, *t*, *r*) with confidence intervals, effect sizes, degrees of freedom and *P* value noted<br>*Give P values as exact values whenever suitable.*
☒	☐	For Bayesian analysis, information on the choice of priors and Markov chain Monte Carlo settings
☒	☐	For hierarchical and complex designs, identification of the appropriate level for tests and full reporting of outcomes
☐	☒	Estimates of effect sizes (e.g. Cohen's *d*, Pearson's *r*), indicating how they were calculated

Our web collection on statistics for biologists contains articles on many of the points above.

Software and code

Policy information about availability of computer code

Data collection	We used Illumina Casava (v.2.19) for the sequencing data and used prefetch (v.2.8.0) to download datasets from GEO sessions.
Data analysis	We used bwa (v.0.7.12), picard-tools (v.1.95), BEDtools (v.2.17.0) and MACS2 (v.2.2.7.1) for mapping and analysis of ChIP-seq analysis. ATAC-seq reads were aligned using bwa (v.0.7.12), reads were then filtered to exclude PCR duplicates using picard-tools (v.1.95). STAR (v.2.4.0h72) and featureCounts (v.2.0.1) were used for gene expression calculation. IDRs of proteins are predicted by PONDR (VSL2 algorithm), Metapredict V2 and MobiDB (v.4.1.0). DISPbind (v.1.0.2, https://github.com/rdong08/DISPbind) and MACS2 (v.2.2.7.1) was used for the DisP-seq data processing. HOMER (v.4.7) was used for motif analysis. The density signals from sequencing data were quantitated using python package pyBigWig (v.0.3.18).

For manuscripts utilizing custom algorithms or software that are central to the research but not yet described in published literature, software must be made available to editors and reviewers. We strongly encourage code deposition in a community repository (e.g. GitHub). See the Nature Portfolio guidelines for submitting code & software for further information.

Data

Policy information about availability of data

All manuscripts must include a data availability statement. This statement should provide the following information, where applicable:

- Accession codes, unique identifiers, or web links for publicly available datasets
- A description of any restrictions on data availability
- For clinical datasets or third party data, please ensure that the statement adheres to our policy

The data generated in this study have been deposited at the Gene Expression Omnibus (GEO) under the series accession number GSE190963 which contains the

datasets GSE190959 (ATAC-seq), GSE190960 (ChIP-seq), GSE190961 (DisP-Seq) and GSE190962 (RNA-seq). We also used the following publicly available data for analysis: SKNMC HiC (GSE105914), SKNMC shGFP and shEWS-FLI1 RNA-seq (GSE61953) and SKNMC histone ChIP-seq (GSE61953).

Field-specific reporting

Please select the one below that is the best fit for your research. If you are not sure, read the appropriate sections before making your selection.

☒ Life sciences ☐ Behavioural & social sciences ☐ Ecological, evolutionary & environmental sciences

For a reference copy of the document with all sections, see nature.com/documents/nr-reporting-summary-flat.pdf

Life sciences study design

All studies must disclose on these points even when the disclosure is negative.

Sample size	DisP-seq experiments were performed in each cell line with two replicates. RNA-seq was conducted with two independent samples with two replicates. Sample sizes for sequencing data were in the same range as similar functional genomic studies.
Data exclusions	No data was exclusion from our analysis.
Replication	Each DisP-seq and RNA-seq experiment was performed twice. All replicates for DisP-seq and RNA-seq were succesful. ChIP-seq and ATAC-seq, were used to generate single profiles to confirm DisP-seq data.
Randomization	Randomization was not relevant to this study because experiments were designed to compare two conditions in the same cell line or to determine the differences between specific cell lines.
Blinding	Blinding was not required for this study. All data collection and analyses were performed using standard sample preparation and bioinformatic pipelines in which all samples were treated equally.

Reporting for specific materials, systems and methods

We require information from authors about some types of materials, experimental systems and methods used in many studies. Here, indicate whether each material, system or method listed is relevant to your study. If you are not sure if a list item applies to your research, read the appropriate section before selecting a response.

Materials & experimental systems

n/a	Involved in the study	
☐	☒	Antibodies
☐	☒	Eukaryotic cell lines
☒	☐	Palaeontology and archaeology
☒	☐	Animals and other organisms
☒	☐	Human research participants
☒	☐	Clinical data
☒	☐	Dual use research of concern

Methods

n/a	Involved in the study	
☐	☒	ChIP-seq
☒	☐	Flow cytometry
☒	☐	MRI-based neuroimaging

Antibodies

Antibodies used	anti-FLI1 (5 µg for ChIP-seq), Abcam, ab15289, lot# GR3229309-6; anti-FLI1 (2 µg for IP and 1:1000 for WB), Abcam, ab133485, lot# GR312646-2; anti-H3K27ac (5 µg for ChIP-seq), ACTIVE MOTIF, 39133, lot# 31521015; anti-AP-2α (5 µg for ChIP-seq and 1:1000 for WB), Santa Cruz, sc-12726X, lot# H1220; anti-NFIB (5 µg for ChIP-seq and 1:1000 for WB), Sigma, HPA003956, lot# 000003516; anti-NFIB (2 µg for IP), Active Motif, 39091, lot# 28915014; anti-H3K9me3 (5 µg for ChIP-seq), Abcam, ab8898, lot# GR232099-3; anti-V5 (5 µg for ChIP-seq and 1:1000 for WB), Cell Signaling, 13202, lot# 6; anti-HA (2 µg for IP and 1:1000 for WB), Roche, 11867423001, lot# 60789700; anti-GABPα (1:1000 for WB), Santa Cruz, sc-22810, lot# B2009; anti-GAPDH (1:10000 for WB), Millipore, MAB374, lot# 3855179; goat anti-rabbit immunoglobulin G-horseradish peroxidase-conjugated (1:10000 for WB), BIO-RAD, 1706515, lot# 350000602; goat anti-mouse immunoglobulin G-horseradish peroxidase-conjugated (1:10000 for WB), BIO-RAD, 1706516, lot# 350000658.
Validation	All the antibodies were validated for the specific application by the manufacturer, tested by independent investigators (from published literature) or provided in this study.<br>ChIP-seq:<br>1. anti-FLI1, https://www.abcam.com/fli1-antibody-ab15289.html, doi:10.1016/j.celrep.2021.109254<br>2. anti-H3K27ac, https://www.activemotif.com/catalog/details/39133/histone-h3-acetyl-lys27-antibody-pab#, doi:10.1016/j.ccell.2014.10.004.<br>3. anti-AP-2α, https://www.scbt.com/p/ap-2alpha-antibody-3b5, doi: 10.1038/s41467-022-29910-4.<br>4. anti-NFIB, https://www.sigmaaldrich.com/US/en/product/sigma/hpa003956, doi: 10.1038/s41467-021-22500-w.<br>5. anti-H3K9me3, https://www.abcam.com/histone-h3-tri-methyl-k9-antibody-chip-grade-ab8898.html, doi: 10.1038/

s41467-022-33147-6.
6. anti-V5, https://www.cellsignal.com/product/productDetail.jsp?productId=13202&utm_medium=b2b&utm_campaign=general, doi: 10.1038/s41467-022-29910-4.
Immunoprecipitation (IP):
1. anti-FLI1, https://www.abcam.com/fli1-antibody-epr4646-ab133485.html, doi: 10.1371/journal.pone.0269077.
2. anti-NFIB, https://www.activemotif.com/catalog/details/39091/nf-1b2-antibody-pab, validated in this study.
3. anti-HA, https://www.sigmaaldrich.com/US/en/product/roche/roahaha, validated in this study.

Eukaryotic cell lines

Policy information about cell lines

Cell line source(s)	Cell lines used in the study included SKNMC (ATCC, HTB-10), HEK293T (ATCC, CRL-3216), MRC5 (ATCC, CCL-171), NCI-H446 (ATCC, HTB-171) and 293T Lenti-X cells (Takara, 632180) .
Authentication	SKNMC, MRC5 and HEK293T cells were authenticated by STR profiling from ATCC. Others were not authenticated.
Mycoplasma contamination	Cells were negative for mycoplasm.
Commonly misidentified lines (See ICLAC register)	No commonly misidentified cell lines were used in the study.

ChIP-seq

Data deposition

☒ Confirm that both raw and final processed data have been deposited in a public database such as GEO.

☒ Confirm that you have deposited or provided access to graph files (e.g. BED files) for the called peaks.

Data access links
May remain private before publication.

www.ncbi.nlm.nih.gov/geo/query/acc.cgi?acc=GSE190960

Files in database submission

H446_H3K27ac
H446_Input
H446_NFIB
H446_shGFP_H3K27ac
H446_shGFP_Input
H446_shGFP_NFIB
H446_shNFIB_H3K27ac
H446_shNFIB_Input
H446_shNFIB_NFIB
SKNMC_H3K9me3
SKNMC_NFIB
SKNMC_EV_H3K27ac
SKNMC_EV_Input
SKNMC_EV_V5
SKNMC_NFIB_del_IDR_H3K27ac
SKNMC_NFIB_del_IDR_Input
SKNMC_NFIB_del_IDR_V5
SKNMC_NFIB_wt_H3K27ac
SKNMC_NFIB_wt_Input
SKNMC_NFIB_wt_V5
SKNMC_shGFP_EV_V5
SKNMC_shGFP_NFIB_WT_V5
SKNMC_shGFP_NFIB_del_DBD_V5
SKNMC_shFLI1_EV_V5
SKNMC_shFLI1_NFIB_WT_V5
SKNMC_shFLI1_NFIB_del_DBD_V5
SKNMC_shFLI1_NFIB
SKNMC_shGFP_NFIB
SKNMC_TFAP2
SKNMC_shFLI1_EV_V5
SKNMC_shFLI1_NFIB_del_DBD_V5
SKNMC_shFLI1_NFIB_WT_V5
SKNMC_shGFP_EV_V5
SKNMC_shGFP_NFIB_del_DBD_V5
SKNMC_shGFP_NFIB_WT_V5

Genome browser session
(e.g. UCSC)

IGV: https://tinyurl.com/ycqtx33d

Methodology

Replicates

Replication is described in Extended Data Table 2.

Sequencing depth

sample total_reads unique_mapped_reads read_length reads_type
H446_H3K27ac 23,964,097 23,333,036 50 single
H446_Input 31,089,317 29,957,059 50 single
H446_NFIB 21,457,211 20,719,572 50 single
H446_shGFP_H3K27ac 17,384,013 16,582,005 50 single
H446_shGFP_Input 25,973,097 24,195,194 50 single
H446_shGFP_NFIB 27,131,676 25,235,666 50 single
H446_shNFIB_H3K27ac 22,737,761 20,266,517 50 single
H446_shNFIB_Input 28,303,994 26,733,514 50 single
H446_shNFIB_NFIB 22,655,651 21,284,507 50 single
SKNMC_H3K9me3 22,692,913 20,903,352 50 single
SKNMC_NFIB 28,070,268 26,744,695 50 single
SKNMC_EV_H3K27ac 26,159,369 25,392,232 50 single
SKNMC_EV_Input 35,676,203 34,398,534 50 single
SKNMC_EV_V5 21,997,892 21,206,625 50 single
SKNMC_NFIB_del_IDR_H3K27ac 35,805,713 34,687,135 50 single
SKNMC_NFIB_del_IDR_Input 33,196,769 32,075,823 50 single
SKNMC_NFIB_del_IDR_V5 24,969,243 24,060,123 50 single
SKNMC_NFIB_wt_H3K27ac 39,662,048 38,411,874 50 single
SKNMC_NFIB_wt_Input 40,205,697 38,699,376 50 single
SKNMC_NFIB_wt_V5 54,890,317 53,066,533 50 single
SKNMC_shFLI1_NFIB 33,698,208 32,760,738 50 single
SKNMC_shGFP_NFIB 34,031,426 32,976,519 50 single
SKNMC_TFAP2 28,984,786 27,528,314 50 single
SKNMC_shFLI1_EV_V5 22,085,722 16,533,099 50 single
SKNMC_shFLI1_NFIB_del_DBD_V5 23,830,650 21,998,080 50 single
SKNMC_shFLI1_NFIB_WT_V5 20,193,770 18,756,355 50 single
SKNMC_shGFP_EV_V5 20,077,179 11,836,875 50 single
SKNMC_shGFP_NFIB_del_DBD_V5 18,708,238 16,990,702 50 single
SKNMC_shGFP_NFIB_WT_V5 20,439,869 17,676,153 50 single

Antibodies

anti-FLI1, Abcam, ab15289; anti-H3K27ac, ACTIVE MOTIF, 39133; anti-AP2a, Santa Cruz, sc-12726X; anti-NFIB, Sigma, HPA003956; anti-H3K9me3, Abcam, ab8898; anti-V5, Cell Signaling, 13202.

Peak calling parameters

```
macs2 callpeak --nomodel -B --SPMR -g hs -q 0.01 -n name -t treatment.bam -c control.bam --outdir callpeaks
bedtools subtract -a macs_peaks.narrowPeak -b hg19-blacklist.bed -A > macs_peaks_rmblacklist.bed
```

Data quality

sample 5_fole_enrichment FDR 5%
H446_H3K27ac 10057 73201
H446_NFIB 37450 61984
H446_shGFP_H3K27ac 14495 68474
H446_shGFP_NFIB 46941 72717
H446_shNFIB_H3K27ac 15645 77273
H446_shNFIB_NFIB 31463 50997
SKNMC_H3K9me3 86 107408
SKNMC_NFIB 41056 50881
SKNMC_EV_H3K27ac 13657 56586
SKNMC_NFIB_del_IDR_H3K27ac 11651 51238
SKNMC_NFIB_del_IDR_V5 7017 9830
SKNMC_NFIB_wt_H3K27ac 12430 59454
SKNMC_NFIB_wt_V5 27609 45977
SKNMC_shFLI1_NFIB 80473 109764
SKNMC_shGFP_NFIB 46818 61529
SKNMC_TFAP2 22992 26500
SKNMC_shFLI1_NFIB_del_DBD_V5 8119 12258
SKNMC_shFLI1_NFIB_WT_V5 35929 67535
SKNMC_shGFP_NFIB_del_DBD_V5 6258 7551
SKNMC_shGFP_NFIB_WT_V5 9455 13833

Software

We used BWA v.0.7.12 to map the sequenced reads, picard-tools v.1.95 to remove duplicates and MACS2 v.2.2.7.1 to call peaks.
