# Supplementary material for: DisP-seq reveals the genome-wide functional organization of DNA-associated disordered proteins
Source: Nat Biotechnol. 2023 Apr 10;42(1):52–64. doi: 10.1038/s41587-023-01737-4 (PMC10791585; doi:10.1038/s41587-023-01737-4)

Original blots related to Fig. 2b

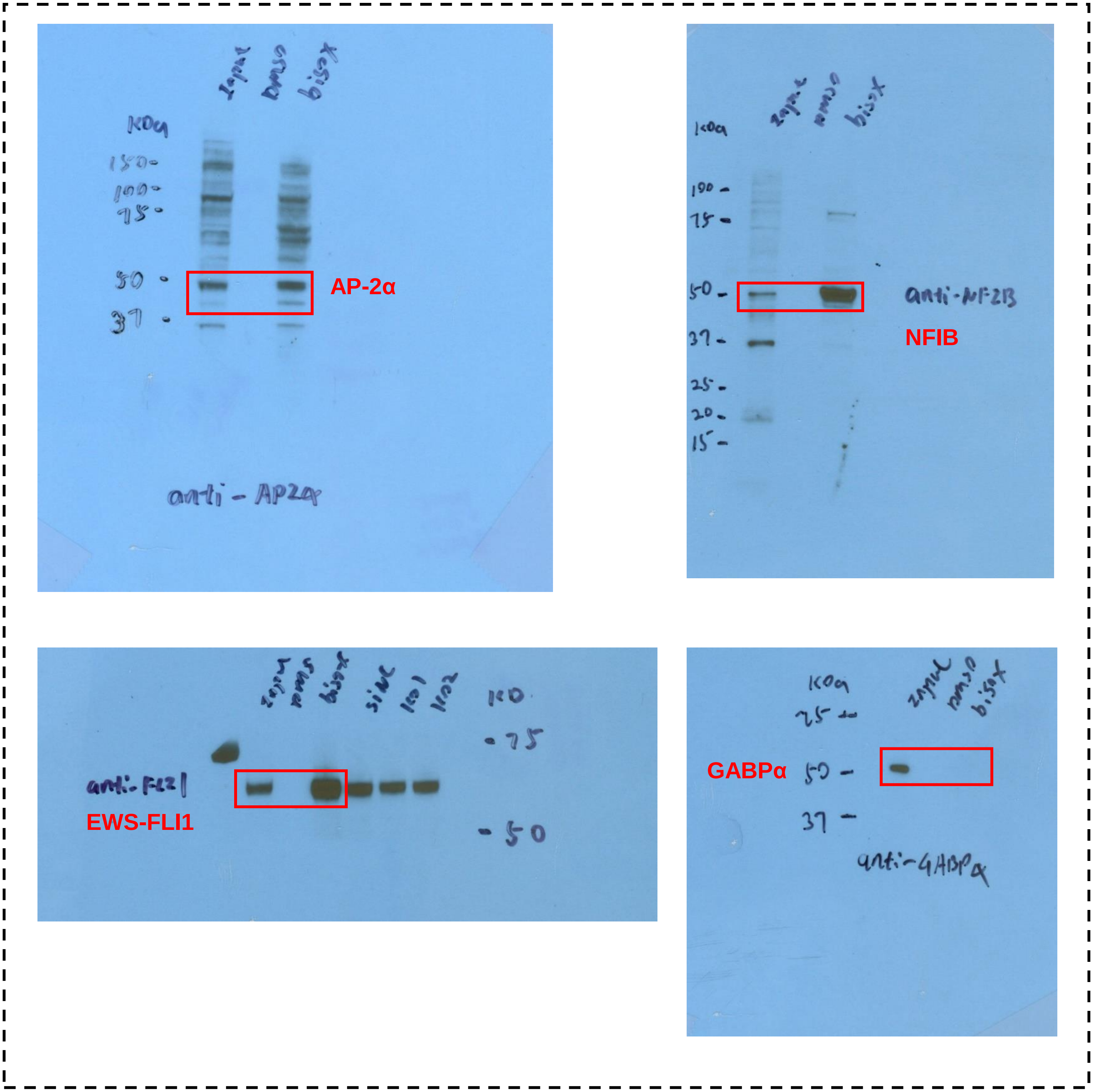

Original blots related to Fig. 4a

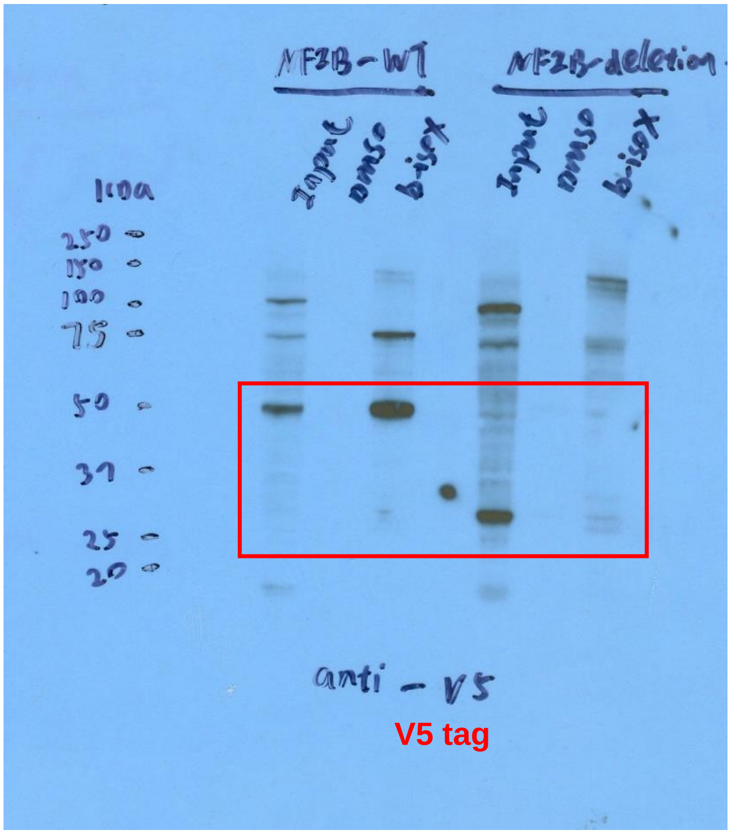

Original blots related to Extended data Fig. 4b

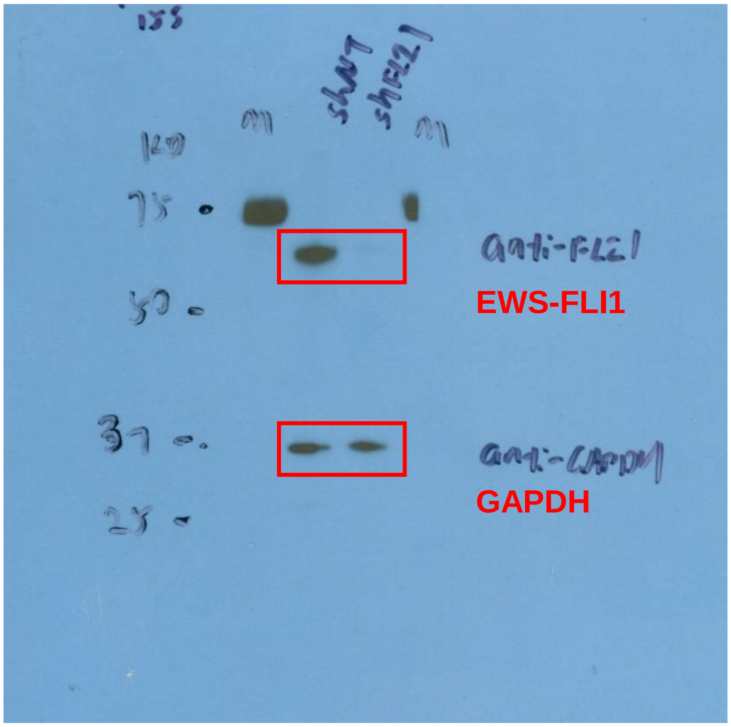

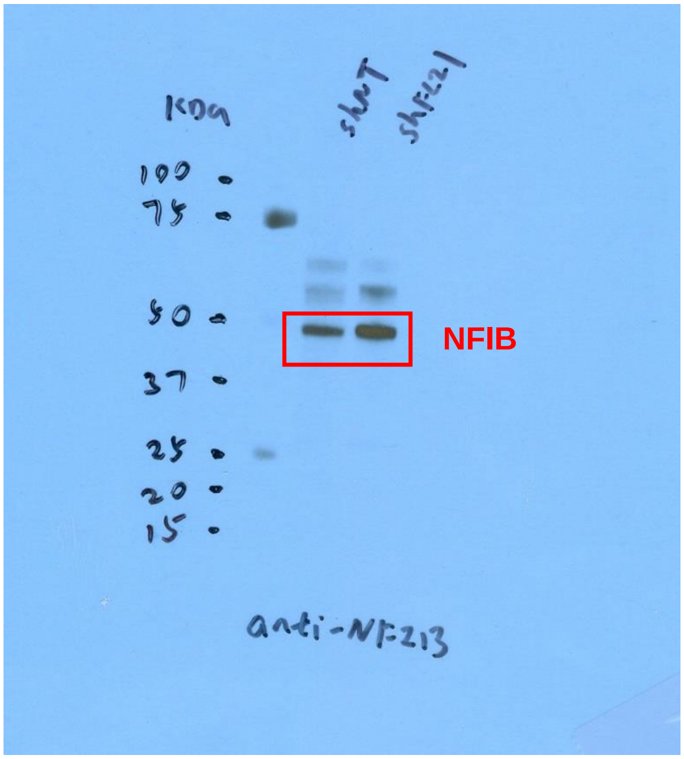

Original blots related to Extended data Fig. 4e

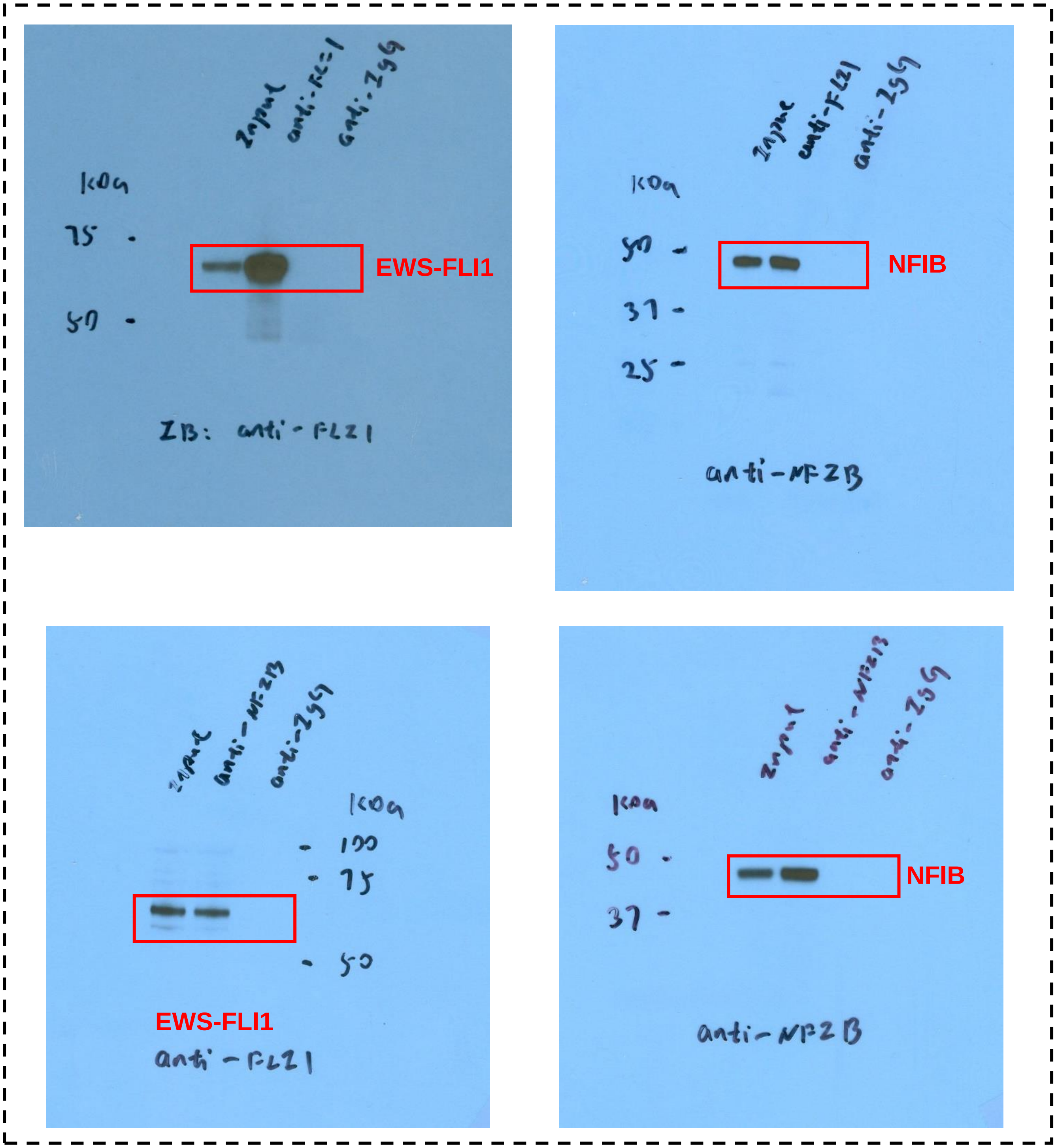

Original blots related to Extended data Fig. 5a

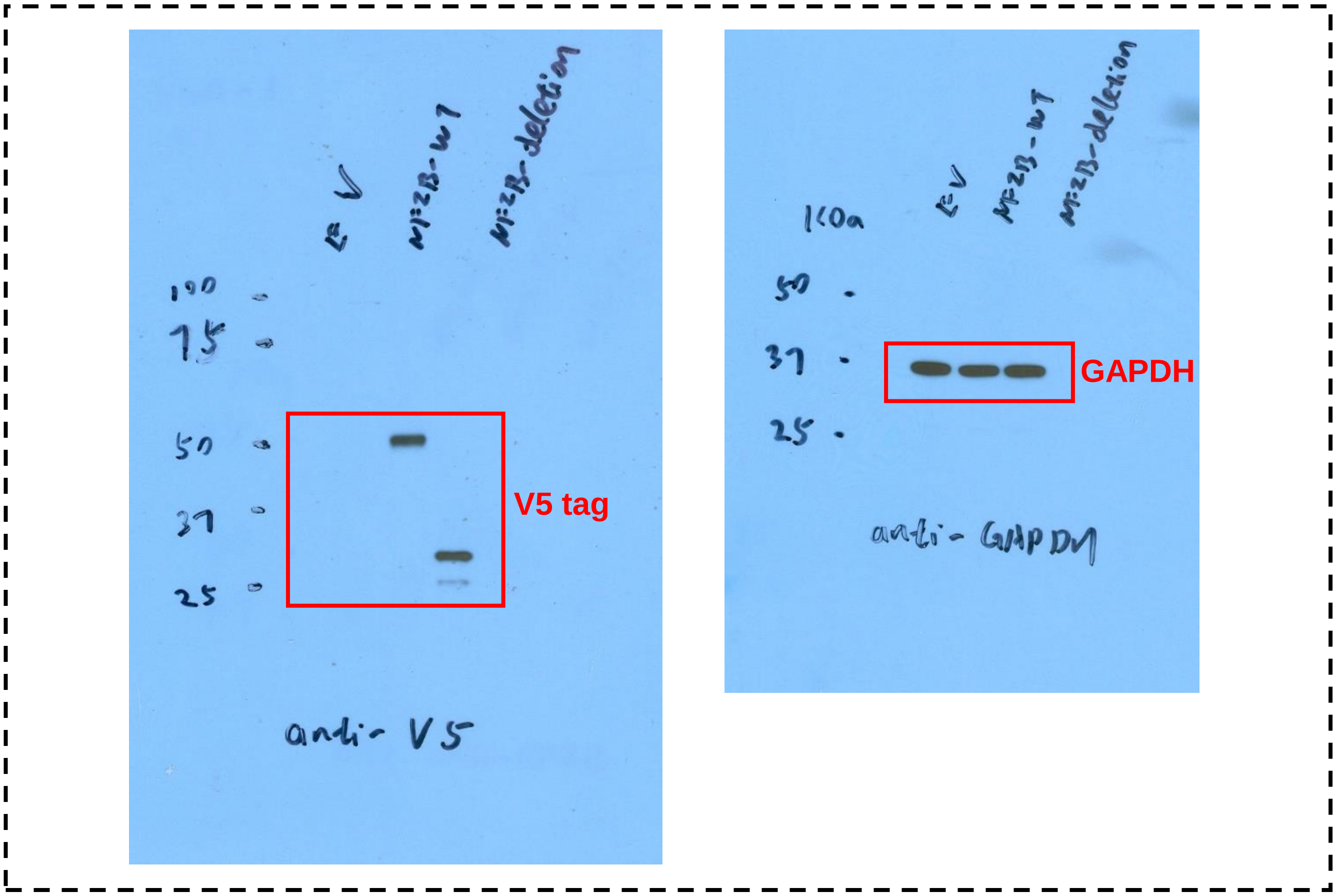

Original blots related to Extended data Fig. 5f

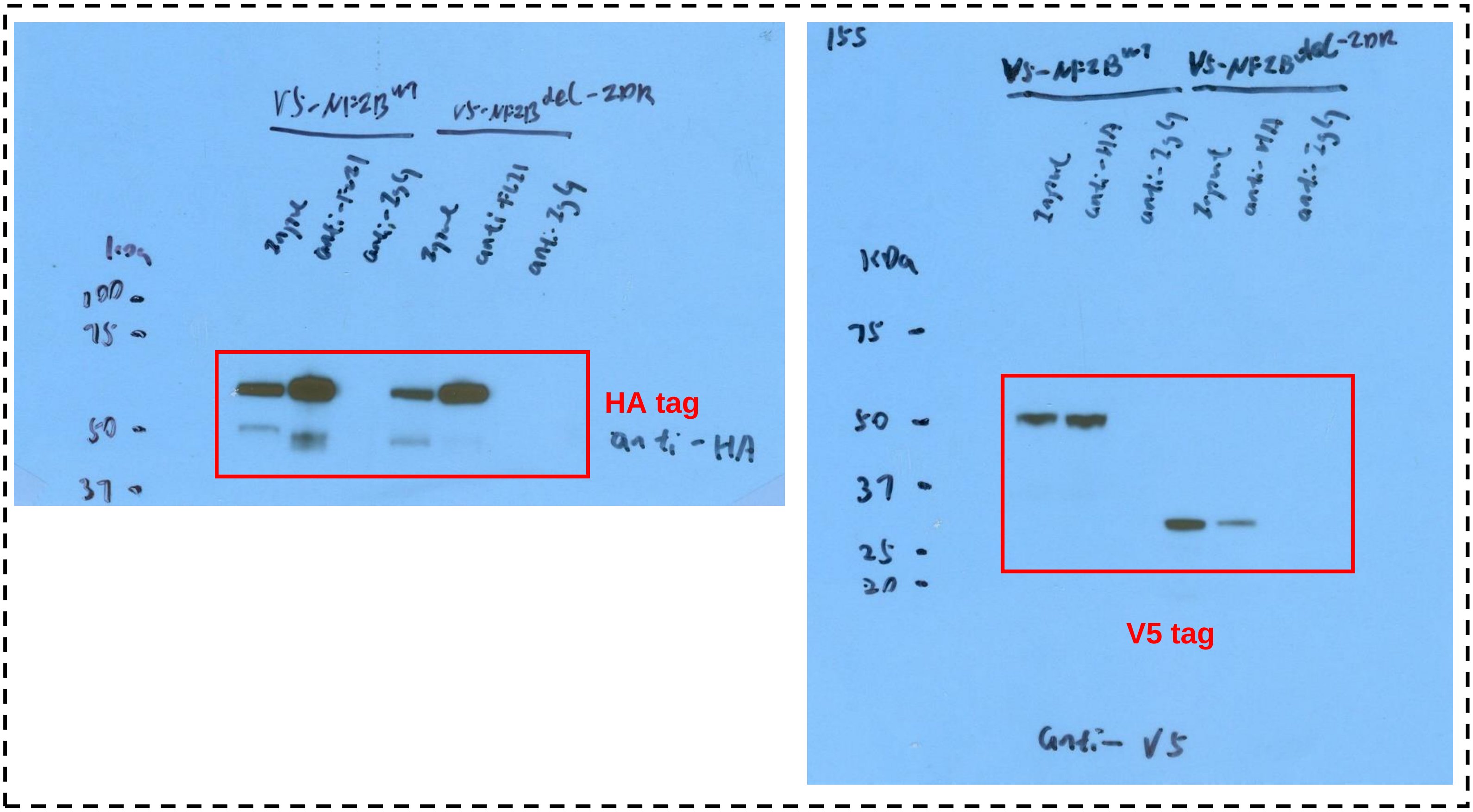

Original blots related to Extended data Fig. 5g

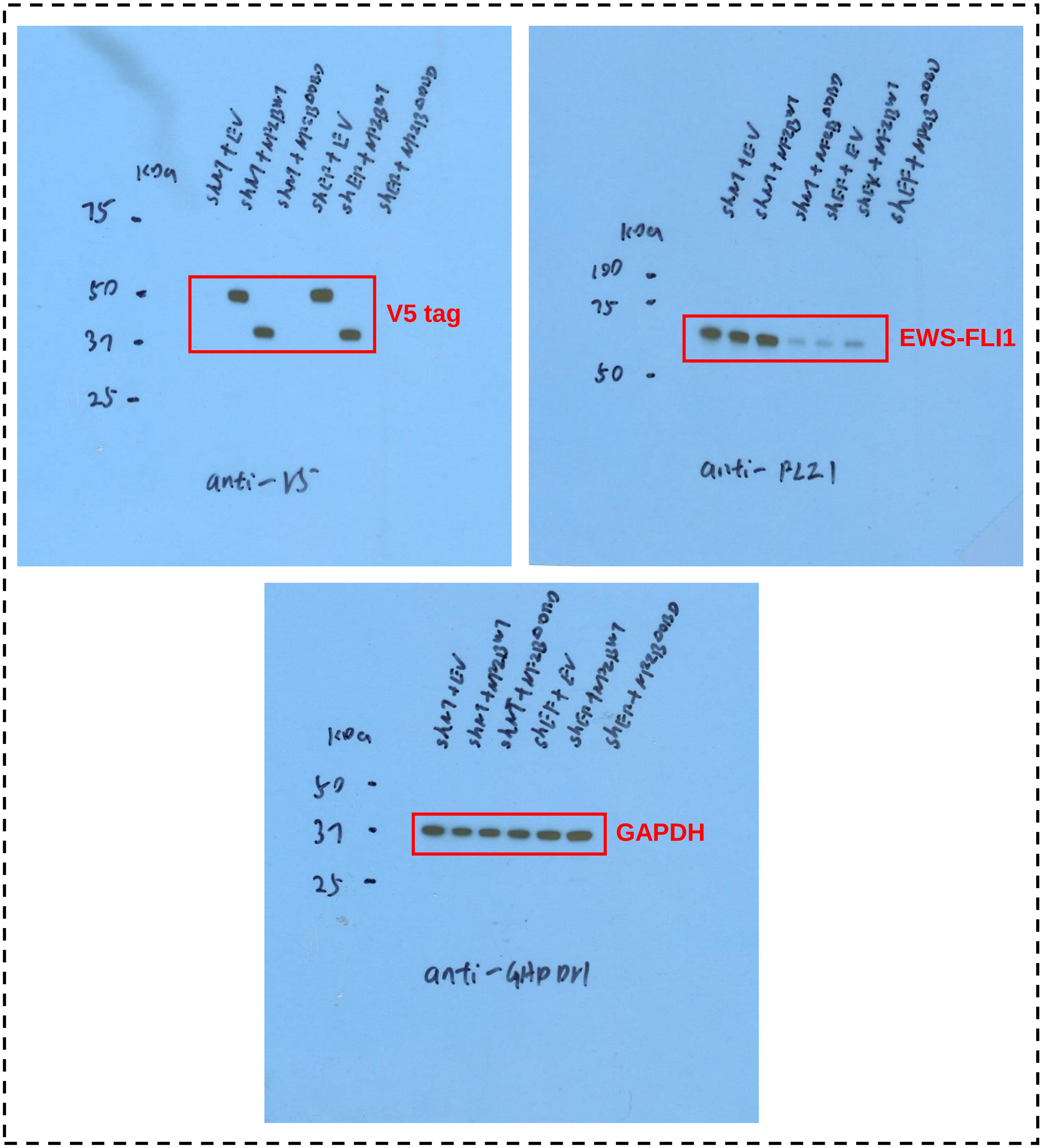

Original blots related to Extended data Fig. 6e

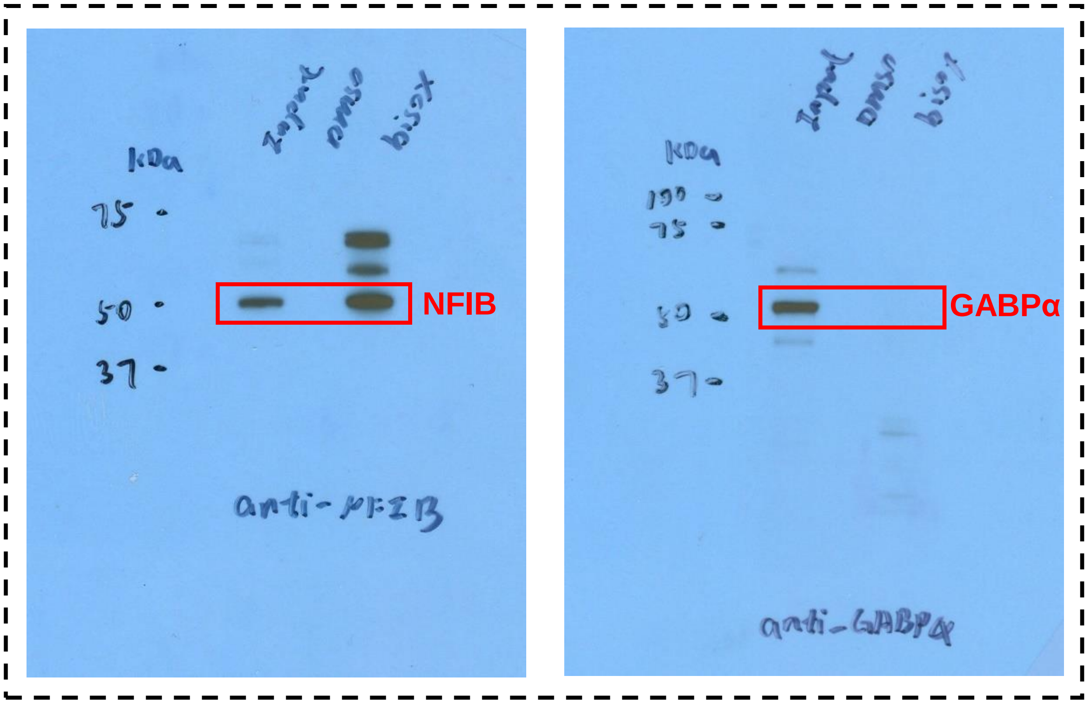

Original blots related to Extended data Fig. 6f

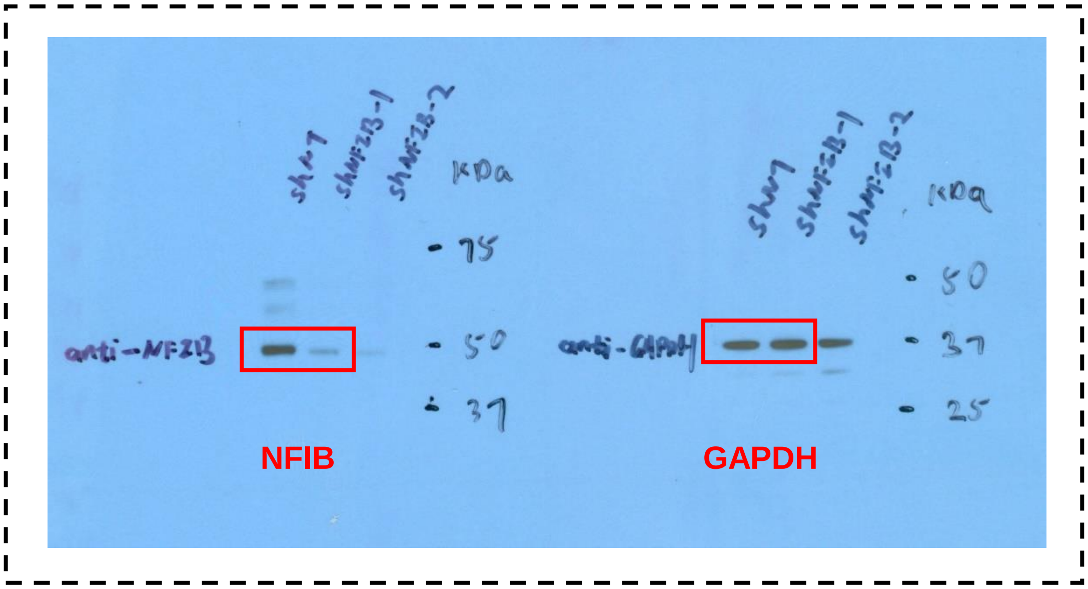

Supplement: Supplementary file 2 — Unprocessed western blots. [file 41587_2023_1737_MOESM2_ESM.pdf]
